# Causes of death after laryngeal cancer diagnosis: A US population-based study

**DOI:** 10.1007/s00405-022-07730-y

**Published:** 2022-11-10

**Authors:** Abdelrahman Yousry Afify, Mohamed Hady Ashry, Mohammed Ahmed Sadeq, Mohamed Elsaid

**Affiliations:** 1 School of Medicine, New Giza University (NGU), Giza, Egypt; 2 Faculty of Medicine, Misr University for Science and Technology, 6th of October, Giza, Egypt; 3 Medical Research Platform, Cairo, Egypt

**Keywords:** Causes of death, Cancer, Laryngeal cancer, Survivorship, Prognosis

## Abstract

**Background:**

Several reports examined the survival of laryngeal cancer (LC) patients, most of these studies only focused on the prognosis of the disease, and just a small number of studies examined non-cancer-related causes of death. The objective of the current study is to investigate and quantify the most common causes of deaths following LC diagnosis.

**Methods:**

The data of 44,028 patient with LC in the United States diagnosed between 2000 and 2018 were retrieved from the Surveillance, Epidemiology, and End Results (SEER) program and analyzed. We stratified LC patients according to various demographic and clinical parameters and calculated standardized mortality ratios (SMRs) for all causes of death.

**Results:**

Over the follow-up period, 25,407 (57.7%) deaths were reported. The highest fatalities (11,121; 43.8%) occurred within 1–5 years following LC diagnosis. Non-cancer causes of death is the leading cause of death (8945; 35.2%), followed by deaths due to laryngeal cancer (8,705; 34.3%), then other cancers deaths (7757; 30.5%). The most common non-cancer causes of death were heart diseases (*N* = 2953; SMR 4.42), followed by other non-cancer causes of death (*N* = 1512; SMR 3.93), chronic obstructive pulmonary diseases (*N* = 1420; SMR 4.90), then cerebrovascular diseases (*N* = 547; SMR 4.28). Compared to the general population, LC patients had a statistically significant higher risk of death from all reported causes.

**Conclusions:**

Non-cancer causes of death is the leading cause of death in LC patients, exceeding deaths attributed to LC itself. These findings provide important insight into how LC survivors should be counselled regarding future health risks.

**Supplementary Information:**

The online version contains supplementary material available at 10.1007/s00405-022-07730-y.

## Introduction

Laryngeal cancer (LC) is one of the most prevalent head and neck malignancies in the United States (US), with an estimated number of 12,470 newly diagnosed cases and 3820 estimated deaths in 2022 [1]. Globally, there were over 209,149 reported LC cases in 2019. LC was also responsible for 123,356 deaths and accounted for 3.26 million DALYs [2].

Studies have demonstrated that the majority of LC tumors are squamous cell carcinomas in nature. Other histological subtypes include adenocarcinomas, sarcomas, lymphomas, and neuroendocrine tumors. Several risk factors, such as smoking and alcohol intake, have been directly associated with the risk of LC development. However, unlike most head and neck tumors that are associated with human papillomavirus infection (HPV), the link between LC and HPV remains controversial [3, 4].

The treatment regimens of LC are mainly comprised of surgery, chemotherapy, and radiotherapy. Early disease stages are usually treated successfully with larynx-conserving surgery alone or local radiotherapy. Advanced disease stages are managed with combination therapy with total laryngectomy, showing survival benefits in patients with T4 disease [4]. Despite the recent advancements in the field, new treatment modalities did not increase the survival rates of LC patients. A recent study analyzed the survival trends of LC from 2004 to 2016, demonstrating a minimal non-significant improvement in both 2- and 5-year relative and overall survival, highlighting the need for further research on LC survivorship [5].

Although determining the causes of mortality after a cancer diagnosis is critical in evaluating treatment efficacy and guiding screening and healthcare policies in cancer survivors, the literature on the causes of death after an LC diagnosis is scarce. To our knowledge, only a few studies have evaluated other non-LC causes of mortality, such as deaths attributed to the development of second primary tumors and suicide. Moreover, the majority of these studies remain fundamentally outdated [6].

In this retrospective population-based study, we utilized the Surveillance, Epidemiology, and End Results (SEER) database to perform a detailed long‐term analysis of the causes of death among LC patients with respect to various demographic and clinicopathological criteria. We further compare the risk of each mortality cause with the US general population.

## Methods

### Study design

We conducted a retrospective, observational cohort in accordance with the Strengthening the Reporting of Observational Studies in Epidemiology (STROBE) guidelines [7].

### Data source and study population

The Surveillance, Epidemiology, and End Results (SEER) program was used to carry out the study [8]. Using the SEER*Stat software, we extracted the data of cancer patients from 2000 to 2018 from the SEER 18 registries, which covers approximately (27.8%) of the US population [9]. An institutional review board approval was not required for this study, because the SEER data are anonymized and considered non-human participant research.

### Study population

We included patients with a confirmed diagnosis of malignant LC with histologic confirmation between 2000 and 2018 in the United States and followed them until death or the end of the specified period. To minimize the risk of selection bias, we included all eligible patients with LC documented in the SEER registries.

### Study outcomes

For the LC patients in our cohort, we analyzed causes of death with respect to different demographical and tumor characteristics, including sex, race, age, and stage at diagnosis and treatment modalities. We further analyzed the causes of death with consideration to different latency periods from the time of diagnosis till death to detect temporal changes with survival time. We stratified causes of death into four distinct periods; up to 1 year, 1–5 years, 5–10 years, and 10 years after LC diagnosis. Causes of death were determined based on the ICD-10-WHO classification.

### Statistical analysis

We calculated standardized mortality ratios (SMRs) for each cause of death reported for our cohort. SMRs were defined as the observed-to-expected ratio (O/E), where “observed” represents the number of patients with LC who died from any defined cause of death within a specific timeframe, and “expected” represents the number of people who are expected to die from the same cause of death in a demographically similar general population within the same period. General US population mortality data were collected by the national center of health statistics. Using the SEER*Stat software (version 8.4.0.1), we estimated SMRs with 95% confidence intervals (CIs). High mortality risk was considered when the observed mortality events for a specific cause in LC patients were significantly higher than the expected mortality events for the same cause in the general population. All statistical tests were two-sided, and a *p* value of less than 0.05 was considered statistically significant.

## Results

### Baseline characteristics

In this study, we reviewed the data of 44,028 patients diagnosed with laryngeal cancer between 2000 and 2018. More than 90% of the included patients were aged > 50 years; 43.6% (*n* = 19,215) were aged 50–64 years, while 46.8% (*n* = 20,602) were aged > 64 years. Most patients were white (*n* = 35,467; 80.6%), males (*n* = 35,520; 80.7%), and were diagnosed with localized LC (*n* = 23,145; 52.6%). Over the follow-up period, 25,407 (57.7%) deaths were reported, with a mean age at death of 69.96 years. Regarding treatment regimes, the majority of patients received radiotherapy (*n* = 32,995; 74.9%), and a smaller fraction of patients underwent cancer-directed therapy (*n* = 16,653; 37.8%) and chemotherapy (*n* = 13,745; 31.2%). The highest fatalities (11,121; 43.8%) were recorded within 1–5 years following LC diagnosis. Moreover, 7165 (28.2%) patients died in the first year following LC diagnosis, 4,856 (19.1%) patients died within 5–10 years, and 2265 (8.9%) died after more than 10 years. Table 1 enlists the baseline characteristics of patients diagnosed with LC, deceased patients according to the time of death after diagnosis, and the mean age of death in each group, respectively.

**Table 1:** Baseline characteristics of all patients with laryngeal cancer and number of deaths according to the time of death following diagnosis

Characteristic	Total no. of patients	Timing of deaths after diagnosis									
		All deaths		< 1 year		1–5 years		5–10 years		> 10 years	
		No. of patients (%)	Mean age at death, y	No. of patients (%)	Mean age at death, y	No. of patients (%)	Mean age at death, y	No. of patients (%)	Mean age at death, y	No. of patients (%)	Mean age at death, y
Overall	44,028	25,407 (100)	69.96	7165 (28.2)	68.33	11,121 (43.8)	68.10	4856 (19.1)	73.46	2265 (8.9)	76.77
Age at diagnosis, y											
0–49	4211	1765 (100)	49.68	427 (24.2)	46.38	829 (47.0)	48.06	297 (16.8)	52.83	212 (12.0)	58.30
50–64	19,215	10,126 (100)	62.15	2624 (25.9)	58.97	4634 (45.8)	60.63	1923 (19.0)	65.67	945 (9.3)	71.30
> 64	20,602	13,516 (100)	78.46	4114 (30.4)	76.58	5658 (41.9)	77.16	2636 (19.5)	81.47	1108 (8.2)	84.96
Sex											
Male	35,520	20,418 (100)	70.00	5740 (28.1)	68.10	8946 (43.8)	68.17	3873 (19.0)	73.74	1859 (9.1)	76.96
Female	8508	4989 (100)	69.80	1425 (28.6)	69.27	2175 (43.6)	67.83	983 (19.7)	72.38	406 (8.1)	75.91
Race											
White	35,467	20,336 (100)	70.64	5559 (27.3)	69.08	8860 (43.6)	68.78	4032 (19.8)	73.87	1885 (9.3)	77.07
Black	6920	4306 (100)	66.27	1391 (32.3)	64.68	1922 (44.6)	64.72	681 (15.8)	70.44	312 (7.2)	73.86
Asian or Pacific Islander	1459	656 (100)	73.67	186 (28.4)	73.62	292 (44.5)	70.40	117 (17.8)	77.28	61 (9.3)	82.53
American Indian/Alaska Native	182	109 (100)	67.18	29 (26.6)	65.71	47 (43.1)	64.35	26 (23.9)	72.06	7 (6.4)	74.10
Disease stage											
Localized	23,145	11,865 (100)	2.46	1959 (16.5)	3.21	5147 (43.4)	2.66	3164 (26.7)	2.15	1595 (13.4)	1.98
Regional	9305	6331 (100)	8.58	2021 (31.9)	13.29	3055 (48.3)	9.44	931 (14.7)	5.05	324 (5.1)	4.15
Distant	7678	5828 (100)	10.51	2525 (43.3)	20.53	2476 (42.5)	10.55	590 (10.1)	4.30	237 (4.1)	3.96
Tumor grade											
Well-differentiated: Grade 1	5638	2818 (100)	72.35	505 (17.9)	70.28	1206 (42.8)	70.07	721 (25.6)	74.77	386 (13.7)	77.66
Moderately differentiated: Grade 2	19,118	11,451 (100)	69.62	2883 (25.2)	67.55	5188 (45.3)	67.97	2325 (20.3)	72.83	1055 (9.2)	76.30
Poorly differentiated: Grade 3	7353	5136 (100)	68.39	1696 (33.0)	67.48	2353 (45.8)	66.97	771 (15.0)	71.95	316 (6.2)	75.20
Undifferentiated, anaplastic: Grade 4	304	222 (100)	69.56	78 (35.1)	68.02	95 (42.8)	67.32	35 (15.8)	75.93	14 (6.3)	77.28
Cancer-directed surgery											
Yes	16,653	8799 (100)	70.46	1667 (18.95)	67.48	4172 (47.41)	68.38	1951 (22.17)	74.03	1009 (11.47)	77.12
Radiotherapy											
Yes	32,995	18,612 (100)	69.70	3825 (20.6)	67.43	9020 (48.5)	67.74	3981 (21.4)	73.21	1786 (9.6)	76.61
Chemotherapy											
Yes	13,745	8709 (100)	65.49	2378 (27.3)	64.34	4462 (51.2)	64.27	1376 (15.8)	69.05	493 (5.7)	72.08

### Causes of death within 1 year after LC diagnosis

**Fig. 1:**
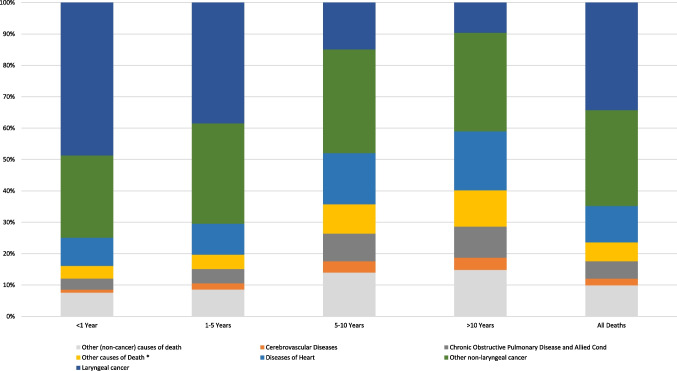
Illustration of causes of death in each latency period following laryngeal cancer diagnosis. *Deaths coded in the SEER database as “other causes of death”

In total, 7,165 patients died within the first year of their diagnosis, with LC accounting for 3,489 deaths (48.7%), other (non-LC) malignancies accounting for 1,877 deaths (26.2%), and non-cancer causes accounting for 1,799 deaths (25.1%). The leading non-cancer cause of death in this period was diseases of the heart (644 deaths; 9.0%) (SMR 6.42; 95% CI 5.93–6.93), followed by other causes of death (290 deaths; 4.05%) (SMR 5.65; 95% CI 5.02–6.34), chronic obstructive pulmonary disease (COPD) (253 deaths; 3.5%) (SMR 6.06; 95% CI 5.33–6.85), and accidents and adverse effects (75 deaths; 1%) (SMR 7.95; 95% CI 6.26–9.97) (Table 2, Fig. 1). Compared to the general population, LC patients had a statistically significant higher risk of death from all reported causes except for Alzheimer’s disease (SMR 0.95; 95% CI 0.44–1.81), tuberculosis (SMR 25.96; 95% CI 0.66–144.63), and deaths from congenital anomalies (SMR 4.9; 95% CI 0.12–27.29).

**Table 2:** Standardized mortality ratios for each cause of death following laryngeal cancer diagnosis

Cause of death	Timing of deaths after diagnosis									
	< 1 year		1–5 years		5–10 years		> 10 years		Total	
	Observed^a^	SMR (95% CI)^b^	Observed^a^	SMR (95% CI)^b^	Observed^a^	SMR (95% CI)^b^	Observed^a^	SMR (95% CI)^b^	Observed^a^	SMR (95% CI)^b^
All causes of death	7165	11.35 (11.09–11.62)^c^	11,121	7.28 (7.14–7.41)^c^	4856	5.20 (5.06–5.35)^c^	2265	6.96 (6.68–7.25)^c^	25,407	7.43 (7.34–7.52)^c^
Laryngeal cancer	3489	19.79 (19.13–20.45)^c^	4276	17.47 (16.95–18)^c^	723	8.47 (7.86–9.11)^c^	217	8.86 (7.72–10.11)^c^	8705	16.39 (16.05–16.74)^c^
Other non-laryngeal cancer causes of death	1877	11.32 (10.81–11.84)^c^	3563	8.80 (8.51–9.09)^c^	1605	6.54 (6.22–6.87)^c^	712	8.65 (8.03–9.31)^c^	7757	8.63 (8.44–8.83)^c^
All non-cancer causes of death	1799	6.22 (5.94–6.52)^c^	3282	3.74 (3.61–3.87)^c^	2528	4.20 (4.04–4.36)^c^	1336	6.11 (5.79–6.45)^c^	8945	4.50 (4.41–4.59)^c^
In situ, benign or unknown behavior neoplasm	24	7.07 (4.53–10.52)^c^	57	6.28 (4.76–8.14)^c^	24	4.21 (2.7–6.27)^c^	19	5.85 (3.52–9.14)^c^	124	5.79 (4.82–6.91)^c^
Tuberculosis	1	25.96 (0.66–144.63)	2	19.01 (2.3–68.66)^c^	0	0 (0–0)	0	0 (0–0)	3	20.87 (4.3–60.99)^c^
Septicemia	70	12.93 (10.08–16.34)^c^	77	6.18 (4.87–7.72)^c^	45	5.05 (3.68–6.76)^c^	29	8.12 (5.44–11.66)^c^	221	7.28 (6.35–8.3)^c^
Other Infectious and Parasitic Diseases including HIV	56	19.97 (15.09–25.94)^c^	53	8.08 (6.05–10.56)^c^	38	9.16 (6.49–12.58)^c^	14	8.90 (4.87–14.93)^c^	161	10.67 (9.09–12.45)^c^
Diabetes Mellitus	38	6.11 (4.32–8.38)^c^	78	3.77 (2.98–4.7)^c^	67	3.76 (2.91–4.77)^c^	44	6.56 (4.76–8.8)^c^	227	4.41 (3.85–5.02)^c^
Alzheimer’s (ICD-9 and 10 only)	9	0.95 (0.44–1.81)	46	1.31 (0.96–1.75)	58	2.29 (1.74–2.97)^c^	32	3.75 (2.57–5.3)^c^	145	1.85 (1.56–2.18)^c^
Diseases of Heart	644	6.42 (5.93–6.93)^c^	1092	3.70 (3.48–3.92)^c^	792	3.98 (3.71–4.27)^c^	425	5.81 (5.27–6.39)^c^	2953	4.42 (4.26–4.58)^c^
Hypertension without Heart Disease	24	5.66 (3.63–8.42)^c^	39	3.25 (2.31–4.45)^c^	31	3.15 (2.14–4.47)^c^	15	5.70 (3.19–9.4)^c^	109	3.80 (3.12–4.58)^c^
Cerebrovascular Diseases	68	3.63 (2.82–4.6)^c^	217	3.78 (3.3–4.32)^c^	174	4.48 (3.84–5.19)^c^	88	6.89 (5.53–8.49)^c^	547	4.28 (3.93–4.66)^c^
Atherosclerosis	7	6.66 (2.68–13.71)^c^	11	3.81 (1.9–6.82)^c^	10	8.31 (3.98–15.27)^c^	0	0 (0–0)	28	5.45 (3.62–7.87)^c^
Aortic Aneurysm and Dissection	9	4.68 (2.14–8.89)^c^	29	4.72 (3.16–6.78)^c^	22	5.20 (3.26–7.87)^c^	10	8.78 (4.21–16.15)^c^	70	5.21 (4.06–6.58)^c^
Other Diseases of Arteries, Arterioles, Capillaries	17	11.67 (6.8–18.68)^c^	24	6.70 (4.29–9.96)^c^	15	6.85 (3.83–11.3)^c^	4	18.63 (5.08–47.71)^c^	60	8.06 (6.15–10.37)^c^
Pneumonia and Influenza	66	5.25 (4.06–6.68)^c^	119	3.29 (2.73–3.94)^c^	83	3.85 (3.07–4.78)^c^	54	6.72 (5.05–8.77)^c^	322	4.11 (3.68–4.59)^c^
Chronic Obstructive Pulmonary Disease and Allied conditions	253	6.06 (5.33–6.85)^c^	513	3.95 (3.62–4.31)^c^	429	4.95 (4.49–5.44)^c^	225	7.13 (6.23–8.13)^c^	1420	4.90 (4.65–5.16)^c^
Stomach and Duodenal Ulcers	7	8.38 (3.37–17.26)^c^	12	4.56 (2.36–7.97)^c^	6	3.95 (1.45–8.59)^c^	2	4.65 (0.56–16.78)	27	4.99 (3.29–7.25)^c^
Chronic Liver Disease and Cirrhosis	33	16.47 (11.34–23.13)^c^	63	11.44 (8.79–14.63)^c^	36	10.17 (7.12–14.08)^c^	12	17.04 (8.81–29.77)^c^	144	12.25 (10.33–14.42)^c^
Nephritis, Nephrotic Syndrome and Nephrosis	26	3.26 (2.13–4.78)^c^	65	2.43 (1.87–3.1)^c^	58	3.48 (2.64–4.5)^c^	24	5.14 (3.3–7.65)^c^	173	3.09 (2.64–3.58)^c^
Complications of Pregnancy, Childbirth, Puerperium	0	0 (0—4067.39)	1	457.03 (11.57–2546.39)^c^	0	0 (0–0)	0	0 (0–0)	1	323.10 (8.18–1800.21)^c^
Congenital Anomalies	1	4.9 (0.12–27.29)	3	4.27 (0.88–12.48)	2	3.9 (0.47–14.11)	1	133.48 (3.38–743.72)^c^	7	4.91 (1.97–10.11)^c^
Symptoms, Signs, and Ill-Defined Conditions	35	7.78 (5.42–10.82)^c^	51	3.17 (2.36–4.17)^c^	51	3.34 (2.49–4.4)^c^	20	4.94 (3.02–7.64)^c^	157	3.94 (3.35–4.6)^c^
Accidents and Adverse Effects	75	7.95 (6.26–9.97)^c^	149	5.24 (4.43–6.15)^c^	99	6.08 (4.94–7.4)^c^	48	11.11 (8.19–14.73)^c^	371	6.35 (5.72–7.03)^c^
Suicide and Self-Inflicted Injury	42	14.32 (10.32–19.35)^c^	63	9.32 (7.16–11.92)^c^	32	11.13 (7.61–15.71)^c^	7	7.76 (3.12–15.98)^c^	144	10.69 (9.01–12.58)^c^
Homicide and Legal Intervention	4	11.53 (3.14–29.52)^c^	11	16.69 (8.33–29.87)^c^	3	13.41 (2.76–39.18)^c^	1	2.22 (0.06–12.39)	19	11.31 (6.81–17.67)^c^
Other Cause of Death	290	5.65 (5.02–6.34)^c^	507	3.10 (2.83–3.38)^c^	453	3.78 (3.44–4.14)^c^	262	5.25 (4.63–5.93)^c^	1512	3.93 (3.73–4.13)^c^

^a^Number of cancer patients who died due to each cause of death

^b^95% Confidence interval

^c^*p* value < 0.05

### Causes of death within 1–5 years after LC diagnosis

In this interval, the total number of deaths has raised, reaching 11,121 patients, of whom 4,276 (38.45%) died due to LC, and 3,563 (30.04%) were due to non-LC malignancies, while non-cancer causes reached 3,282 (29.51%) being the lowest number of deaths. During this time interval, the major non-cancer causes of death were heart diseases, followed by COPD, and other causes of death, with mortality rates reaching 9.82% (*n* = 1092), 4.61% (*n* = 513), and 4.56% (*n* = 507). However, the fourth leading non-cancer cause was cerebrovascular diseases representing 1.95% of deaths (*n* = 217). Moreover, the standardized mortality ratio (SMR) of these causes indicated a statistically significant higher risk of death which reached (SMR 3.70; 95% CI 3.48–3.92) for heart diseases, (SMR 3.95; 95% CI 3.62–4.31) for COPD, (SMR 3.10; 95% CI 2.83–3.38) for other causes of death and (SMR 3.78; 95% CI 3.3–4.32) for cerebrovascular diseases, compared to the general population. In general, all reported causes had a statistically significant higher risk of death compared to the general population except for Alzheimer’s disease (SMR 1.31; 95% CI 0.96–1.75) and congenital anomalies (SMR 4.27; 95% CI 0.88–12.48) that were higher but not significant (Table 2, Fig. 1).

### Causes of death within 5–10 years after LC diagnosis

The number of deaths in this period declined to reach 4,856 patients representing only 19.11% of all deaths. In contrast to the first two intervals, non-cancer causes were responsible for more deaths (*n* = 2528; 52.06%) than cancer causes (*n* = 2328; 47.94%). In detail, LC was the cause of death in 723 (14.89%) patients, representing the second lowest percentage in all four intervals. On the other hand, non-LC malignancies reached 33.05% (*n* = 1605), representing the highest percentage in the four intervals. Similar to 1–5 year interval, heart diseases, other causes of death, COPD, and cerebrovascular diseases were the most common non-cancer causes of death by (792 deaths; 14.6%) (SMR 3.98; 95% CI 3.71–4.27), (453 deaths; 9.33%) (SMR 3.78; 95% CI 3.44–4.14), (429 deaths; 8.83%) (SMR 4.95; 95% CI 4.49–5.44), and (88 deaths; 1.81%) (SMR 6.89; 95% CI 5.53–8.49), respectively (Table 2, Fig. 1).

### Causes of death > 10 years after LC diagnosis

The lowest mortality rate occurred after 10 years of diagnosis, with a total of 2,265 deaths representing only 8.91% of total deaths. In addition, LC, as a cause of death, reached its lowest percentage among all four intervals at 9.58% only (*n* = 217). On the other hand, non-cancer causes reached their highest percentage with 58.98% (*n* = 1336) of all deaths after 10 years of LC diagnosis. The leading non-cancer cause of death remained similar to the previous intervals (5–10 years); heart diseases followed by other causes of death, COPD, then cerebrovascular diseases. The mentioned causes had a statistically significant higher risk of mortality than the general population with (SMR 5.81; 95% CI 5.27–6.39) for heart diseases, (SMR 5.25; 95% CI 4.63–5.93) for other causes of death, (SMR 7.13; 95% CI 6.23–8.13) for COPD, and (SMR 6.89; 95% CI 5.53–8.49) for cerebrovascular diseases. Moreover, all non-cancer causes had a statistically significant higher risk of mortality except for stomach and duodenal ulcers (SMR 4.65; 95% CI 0.56–16.78), and homicide and legal intervention (SMR 2.22; 95% CI 0.06–12.39) that were higher but statistically insignificant, compared to the general population (Table 2, Fig. 1).

### Subgroup analysis

We performed subgroup analysis to assess the association between different baseline characteristics and causes of mortality, including age (Supp. Tables 1–3), gender (Supp. Tables 4 and 5), race (Supp. Tables 6–9), stage of the disease (Supp. Tables 10–12) and treatment (Supp. Tables 13–15). Detailed analysis of different patient demographical and pathological characteristics across the four latency periods revealed an overall similar trend to the general cohort. No substantial differences in the distribution of the top causes of death were found in the examined subgroups, and only variabilities in SMRs were expectedly observed, given the heterogeneous nature of the comparator standard population for each subgroup. When comparing the total number of deaths in each subgroup, non-cancer causes of death were the leading mortality cause in LC patients’ subgroups who were diagnosed later in life, males, whites, received radiotherapy or surgery and in those diagnosed with localized LC. Furthermore, diseases of the heart continued to be the leading non-cancer cause of death in LC patients in all analyses, with COPD, other non-cancer causes of death, and cerebrovascular disease alternating for the highest risks (see Supporting Tables for details on all subgroups).

## Discussion

We analyzed the causes of death of 44,028 laryngeal cancer patients within a range of 18 years from 2000 to 2018. The mortality rate reached 57.7% (*n* = 25,407) of which only 34.26% (*n* = 8705) were because of LC. A total of 16,702 patients died from other causes, representing 65.74% of the mortality rate, further divided into other types of cancer (30.53%) and non-cancer causes (35.2%). LC was responsible for almost half of the deaths (*n* = 3489; 48.7%) within the first year after diagnosis, while it reached 38.45% (*n* = 4276) between 1 and 5 years. However, a tapering effect was observed for the reported causes of death after 5 years of diagnosis, in which a shift toward non-cancer-attributable deaths was noticeable. The percentage dropped dramatically after 5 years from diagnosis representing only 14.89% (*n* = 723) and 9.58% (*n* = 217) in the periods 5–10 years and > 10 years, respectively. In addition, one intriguing finding of the current study is the statistically significant and, in some instances, massive increase in the risk of death compared to the general population, highlighting the vulnerability of LC patients to most causes of death.

Compared to the high incidence of distant metastasis (DM) in patients with lung and breast cancer, LC has a relatively low rate of manifesting as a distant disease at diagnosis. However, it usually has a fatal outcome when it occurs. In our study, 7,678 (17.44%) patients were diagnosed with distant disease, and of those patients, we observed 5,828 (75.91%) deaths, constituting 22.94% of all deaths. We also observed 2,525 (43.3%) deaths within 1 year of diagnosis in this cohort. The reported incidence of distant metastasis in the head and neck squamous cell cancers (HNSCC) is approximately 10–50% [10]. Coca-Pelaz et al. analyzed the data of 443 patients with surgically treated HNSCC and detected a total of 60 (13.5%) patients who had developed DM [11]. This study included 197 LC patients with a lower DM incidence (8%). Spector et al. have also detected a similar rate (8.5%) among 2,550 patients with laryngeal and hypopharyngeal cancers [12]. In another study, the majority of deaths were observed in the local/regional (L/R) recurrence and DM groups, with only a minority of deaths due to second primary malignancies (L/R, 48%; DM, 41%; second primary, 11%) [13].

Second primary cancer in LC patients is a common event that might be explained by the lifestyle choices of these patients and the “field cancerization” hypothesis or the recently proposed second field tumor concept [14, 15]. In our study, we observed 7757 (30.53%) deaths due to other non-laryngeal cancers (SMR 8.63; 95 CI 8.44–8.83). Chu et al. have highlighted factors associated with a higher incidence of a new primary malignancy [16]. These factors included early T-classification, tobacco use, and less index tumor recurrence. Contrary to other cancer sites, radiotherapy does not seem to increase the incidence of second primary cancers; however, it may modify the pattern of tumors in the head and neck region [17]. A previous SEER analysis found that RT for LC was associated with a lower risk of second head and neck primary malignancies [18]. Mortality rates of patients with LC and a second primary tumor seem to depend on the new cancer site. Patients with second primary tumors of the head and neck have lower death rates than those with non-head and neck second primaries [16, 19–21].

The most reported non-cancer causes of death were heart diseases (*n* = 2953) and COPD (*n* = 1420). Both, along with LC, are associated with cigarette smoking. Associations between LC and these diseases were reported in the literature. Research by Mucha-Małecka et al. reported that the percentage of patients with T1N0M0 glottic cancer and ischemic heart disease was 44% in their cohort. The number of patients who had COPD was 44% as well [22]. Two other studies have reported a high prevalence of COPD among patients with LC [23, 24]. Zheng et al. found that the second highest risk of death due to COPD across different cancer sites was laryngeal cancer (SMR 5.54; 95% CI 5.34–5.75) which was very close to our findings (SMR 4.90; 95% CI 4.65–5.16) [25]. Our results, in line with the literature, support the principle of aiming COPD prevention strategies at patients with cancer, particularly LC. Heart disease in LC patients may also occur due to subclinical thyroid disease, since the thyroid tissue is anterior to the larynx and included in the RT treatment [10, 26]. Another factor contributing to heart disease among LC patients is the cardiotoxicity attributed to chemotherapy. Franchin et al. have observed cardiotoxicity in 15 (14%) patients due to chemotherapy. Of those, two patients developed severe symptomatic bradycardia (heart rate < 39 beats/min), and one experienced myocardial ischemia [27].

Our findings also demonstrate high mortality rates attributed to suicide and self-inflicted injury (SMR 10.69; 95% CI 9.01–12.58), with the highest rates occurring within 1 year of diagnosis (SMR 14.32; 95% CI 10.32–19.35). Historically, cancer has been identified as a risk factor for suicide, especially in the short period after diagnosis [28]. Luckily, the risk of suicide steadily decreased from 1960 to 1999 due to improved psychological care and attention to aspects of quality of life. Regardless, the fact remains that the highest suicide rates among cancer patients occur in those with respiratory malignancies, including the larynx. Hem et al. have reported an increased risk of suicide in both males and females. The highest risk was in the first months after the diagnosis and among males with respiratory malignancy [28]. Misono et al. have demonstrated similar findings among SEER registry cancer patients. They have observed that the suicide rate among these patients was double that of the general USA population [29].

Several studies have reported age as an important prognostic factor in laryngeal carcinoma. In our study, 13,516 deaths were in patients 65 years or older, of whom 4114 died within the first year after diagnosis. Reizenstein et al. have also demonstrated that being 80 years or older is associated with an increased risk of death attributed to LC [30]. On the other hand, the survival of stage IV glottic cancer was reportedly better in patients younger than 56 years [12]. Another important commonly described prognostic factor was the grade of the disease. Our study found that patients who died with well-differentiated and poorly differentiated tumors were 2818 (49.98%) and 222 (73.02%), respectively. These findings were similar to the literature. For instance, Johansen et al. reported the overall survival percentage of patients with well, moderately, and poorly differentiated were 70 ± 2%, 60 ± 2%, and 47 ± 3%, respectively [31].

### Strengths and limitation

Although the incidence of laryngeal cancer has decreased over the past decades, the reported improvement in patients’ survival is still minimal, necessitating a detailed analysis of mortality outcomes. The current study provides the most comprehensive and recent population-based, long-term analysis of the causes of death among patients diagnosed with laryngeal cancer with a detailed analysis of different patients’ demographics and tumor characteristics. However, there are several limitations to this study. These limitations include the retrospective nature of the analysis, which may have introduced some inherent bias. We could not account for patients’ comorbidities, the impact of specific treatment regimens, or assess patients’ quality of life, because the database does not report such data. Furthermore, a detailed analysis of death causes coded as “other causes of death” was not possible. Slight misclassification of the causes of death in specific patient groups was reported before; however, the authors concluded that the data are overall reliable and valid [32].

## Conclusions

Combined deaths from LC and non-LC cancers represent the majority of mortality causes after LC diagnosis. However, fatalities from non-cancer causes account for a large percentage of deaths among patients with LC especially with increased survival time. Deaths from heart disease and COPD were among the most prevalent causes of non-LC deaths over the follow-up periods in our cohort. Our cohort also had a statistically significant higher risk of death compared to the general US population across most of the reported causes. These findings shed light on how LC survivors should be counselled and monitored in the future.

## Supplementary Information

Below is the link to the electronic supplementary material.

Supplementary file1 (DOCX 210 KB)

## Data Availability

All analyzed data are provided within the manuscript and its online supplementary material. Public access to the SEER database is available at https://seer.cancer.gov/.

## References

[1] Siegel RL, Miller KD, Fuchs HE, Jemal A (2022). Cancer statistics, 2022. CA Cancer J Clin.

[2] Lin C, Cheng W, Liu X (2022). The global, regional, national burden of laryngeal cancer and its attributable risk factors (1990–2019) and predictions to 2035. Eur J Cancer Care (Engl).

[3] De Stefani E, Correa P, Oreggia F (1987). Risk factors for laryngeal cancer. Cancer.

[4] Steuer CE, El-Deiry M, Parks JR (2017). An update on larynx cancer. CA Cancer J Clin.

[5] Li MM, Zhao S, Eskander A (2021). Stage migration and survival trends in laryngeal cancer. Ann Surg Oncol.

[6] Ferlito A, Haigentz M, Bradley PJ (2014). Causes of death of patients with laryngeal cancer. Eur Arch Oto-Rhino-Laryngol.

[7] von Elm E, Altman DG, Egger M (2014). The Strengthening the Reporting of Observational Studies in Epidemiology (STROBE) Statement: guidelines for reporting observational studies. Int J Surg Lond Engl.

[8] Surveillance Research Program, National Cancer Institute. SEER*Stat software, version 8.4.0.1, Released April 2021

[9] Surveillance, Epidemiology, and End Results (SEER) Program (http://www.seer.cancer.gov). SEER*Stat Database: Incidence - SEER , 18 Registries (excl AK), Nov 2020 Sub (2000–2018) for SMRs—Linked to County Attributes—Total U.S., 1969–2019 Counties

[10] Ferlito A, Haigentz M, Bradley PJ (2014). Causes of death of patients with laryngeal cancer. Eur Arch Otorhinolaryngol.

[11] Coca-Pelaz A, Rodrigo JP, Suárez C (2012). Clinicopathologic analysis and predictive factors for distant metastases in patients with head and neck squamous cell carcinomas. Head Neck.

[12] Spector GJ, Sessions DG, Lenox J (2004). Management of stage IV glottic carcinoma: therapeutic outcomes. Laryngoscope.

[13] Wolf GT, Fisher SG, Hong WK, Hillman R, Spaulding M, Laramore GE, Endicott JW, McClatchey K, Henderson WG (1991). Induction chemotherapy plus radiation compared with surgery plus radiation in patients with advanced laryngeal cancer. N Engl J Med.

[14] Slaughter DP, Southwick HW, Smejkal W (1953). “Field cancerization” in oral stratified squamous epithelium. Clin Implications Multicentric Origin Cancer.

[15] Braakhuis BJM, Tabor MP, Alain Kummer J (2003). A genetic explanation of Slaughter’s concept of field cancerization: evidence and clinical implications. Cancer Res.

[16] Chu P-Y, Chang S-Y, Huang J-L, Tai S-K (2010). Different patterns of second primary malignancy in patients with squamous cell carcinoma of larynx and hypopharynx. Am J Otolaryngol.

[17] Rennemo E, Zätterström U, Evensen J, Boysen M (2009). Reduced risk of head and neck second primary tumors after radiotherapy. Radiother Oncol.

[18] Rusthoven K, Chen C, Raben D, Kavanagh B (2008). Use of external beam radiotherapy is associated with reduced incidence of second primary head and neck cancer: a SEER database analysis. Int J Radiat Oncol.

[19] Erkal HŞ, Mendenhall WM, Amdur RJ (2001). Synchronous and metachronous squamous cell carcinomas of the head and neck mucosal sites. J Clin Oncol.

[20] Farhadieh RD, Salardini A, Yang JL (2010). Diagnosis of second head and neck tumors in primary laryngeal SCC is an indicator of overall survival and not associated with poorer overall survival: a single centre study in 987 patients: primary laryngeal SCC. J Surg Oncol.

[21] León X, Quer M, Diez S (1999). Second neoplasm in patients with head and neck cancer. Head Neck.

[22] Mucha-Małecka A, Małecki K, Amrogowicz N (2021). Prognostic factors in elderly patients with T1 glottic cancer treated with radiotherapy. Sci Rep.

[23] Gottlieb M, Marsaa K, Godtfredsen NS, Mellemgaard A (2015). Prevalence and management of pulmonary comorbidity in patients with lung and head and neck cancer. Acta Oncol.

[24] van de Schans SAM, Janssen-Heijnen MLG, Biesma B (2007). COPD in cancer patients: higher prevalence in the elderly, a different treatment strategy in case of primary tumours above the diaphragm, and a worse overall survival in the elderly patient. Eur J Cancer.

[25] Zhang S-S, Xia Q-M, Zheng R-S, Chen W-Q (2015). Laryngeal cancer incidence and mortality in China, 2010. J Cancer Res Ther.

[26] Gencer B, Collet T-H, Virgini V (2013). Subclinical thyroid dysfunction and cardiovascular outcomes among prospective cohort studies. Endocr Metab Immune Disord-Drug Targets.

[27] Franchin G, Vaccher E, Politi D (2009). Organ preservation in locally advanced head and neck cancer of the larynx using induction chemotherapy followed by improved radiation schemes. Eur Arch Otorhinolaryngol.

[28] Hem E, Loge JH, Haldorsen T, Ekeberg Ø (2004). Suicide risk in cancer patients from 1960 to 1999. J Clin Oncol.

[29] Misono S, Weiss NS, Fann JR (2008). Incidence of suicide in persons with cancer. J Clin Oncol.

[30] Reizenstein JA, Bergström SN, Holmberg L (2009). Impact of age at diagnosis on prognosis and treatment in laryngeal cancer. Head Neck.

[31] Vendelbo Johansen L, Grau C, Overgaard J (2003). Laryngeal carcinoma multivariate analysis of prognostic factors in 1 252 consecutive patients treated with primary radiotherapy. Acta Oncol.

[32] Hu C-Y, Xing Y, JaniceN C, Chang GJ (2013). Assessing the utility of cancer-registry-processed cause of death in calculating cancer-specific survival. Cancer.

