# Supplementary material for: Causes of death after laryngeal cancer diagnosis: A US population-based study
Source: Eur Arch Otorhinolaryngol. 2022 Nov 10;280(4):1855–64. doi: 10.1007/s00405-022-07730-y (PMC9989001; doi:10.1007/s00405-022-07730-y)
Supplement: Supplementary file 1 — Supplementary file1 (DOCX 210 KB) [file 405_2022_7730_MOESM1_ESM.docx]

| **Supplementary Table 1.** Standardized-mortality ratios (SMRs) for each cause of death following laryngeal cancer diagnosis in patients younger than 49 years. | | | | | | | | | | |
| --- | --- | --- | --- | --- | --- | --- | --- | --- | --- | --- |
| **Cause of death** | <1 year | | 1-5 years | | 5-10 years | | >10 years | | Total | |
|  | Observed^1^ | SMR (95% CI^2^) | Observed^1^ | SMR (95% CI^2^) | Observed^1^ | SMR (95% CI^2^) | Observed^1^ | SMR (95% CI^2^) | Observed^1^ | SMR (95% CI^2^) |
| All causes of death | 427 | 68.6 (62.25 - 75.43) ^b^ | 829 | 56.25 (52.48 - 60.21) ^b^ | 297 | 27.4 (24.37 - 30.7) ^b^ | 212 | 37.59 (32.7 - 43.01) ^b^ | 1,765 | 47.14 (44.96 - 49.39) ^b^ |
| Laryngeal cancer | 201 | 81.1 (70.28 - 93.12) ^b^ | 398 | 96.36 (87.12 - 106.3) ^b^ | 65 | 29.38 (22.67 - 37.44) ^b^ | 39 | 42.22 (30.02 - 57.72) ^b^ | 703 | 72.14 (66.9 - 77.67) ^b^ |
| Other non-laryngeal cancer causes of death | 142 | 66.69 (56.17 - 78.6) ^b^ | 270 | 51.37 (45.42 - 57.88) ^b^ | 107 | 28.39 (23.27 - 34.31) ^b^ | 73 | 32.33 (25.34 - 40.65) ^b^ | 592 | 44.14 (40.66 - 47.84) ^b^ |
| All non-cancer causes of death | 84 | 51.97 (41.45 - 64.34) ^b^ | 161 | 30.08 (25.61 - 35.1) ^b^ | 125 | 25.72 (21.41 - 30.65) ^b^ | 100 | 40.69 (33.1 - 49.49) ^b^ | 470 | 32.9 (29.99 - 36.01) ^b^ |
| In situ, benign or unknown behavior neoplasm | 3 | 159.11 (32.81 - 464.98) ^b^ | 3 | 57.62 (11.88 - 168.38) ^b^ | 1 | 22.04 (0.56 - 122.81) | 1 | 57.99 (1.47 - 323.12) ^b^ | 8 | 59.91 (25.86 - 118.05) ^b^ |
| Tuberculosis | 0 | 0 (0 - 0) | 0 | 0 (0 - 0) | 0 | 0 (0 - 0) | 0 | 0 (0 - 0) | 0 | 0 (0 - 0) |
| Syphilis | 0 | 0 (0 - 0) | 0 | 0 (0 - 0) | 0 | 0 (0 - 0) | 0 | 0 (0 - 0) | 0 | 0 (0 - 0) |
| Septicemia | 5 | 68.28 (22.17 - 159.33) ^b^ | 5 | 21.8 (7.08 - 50.88) ^b^ | 3 | 13.73 (2.83 - 40.13) ^b^ | 3 | 44.64 (9.21 - 130.46) ^b^ | 16 | 27.2 (15.55 - 44.17) ^b^ |
| Other Infectious and Parasitic Diseases including HIV | 7 | 71.05 (28.57 - 146.39) ^b^ | 9 | 31.1 (14.22 - 59.04) ^b^ | 6 | 29.78 (10.93 - 64.82) ^b^ | 3 | 46.04 (9.49 - 134.54) ^b^ | 25 | 38.19 (24.72 - 56.38) ^b^ |
| Diabetes Mellitus | 0 | 0 (0 - 282.26) | 1 | 15.93 (0.4 - 88.75) | 1 | 23.06 (0.58 - 128.5) | 2 | 167.44 (20.28 - 604.85) ^b^ | 4 | 30.5 (8.31 - 78.09) ^b^ |
| Alzheimer’s (ICD-9 and 10 only) | 0 | 0 (0 - 0) | 0 | 0 (0 - 0) | 0 | 0 (0 - 0) | 0 | 0 (0 - 0) | 0 | 0 (0 - 0) |
| Diseases of Heart | 23 | 49.36 (31.29 - 74.07) ^b^ | 42 | 27.29 (19.66 - 36.88) ^b^ | 35 | 24.13 (16.8 - 33.55) ^b^ | 30 | 34.78 (23.47 - 49.65) ^b^ | 130 | 30.1 (25.15 - 35.74) ^b^ |
| Hypertension without Heart Disease | 2 | 187.16 (22.67 - 676.09) ^b^ | 1 | 45.43 (1.15 - 253.13) ^b^ | 0 | 0 (0 - 153.5) | 1 | 46.97 (1.19 - 261.7) ^b^ | 4 | 51.27 (13.97 - 131.27) ^b^ |
| Cerebrovascular Diseases | 0 | 0 (0 - 47.28) | 11 | 38.56 (19.25 - 69) ^b^ | 6 | 50.19 (18.42 - 109.25) ^b^ | 4 | 65.94 (17.97 - 168.83) ^b^ | 21 | 38.64 (23.92 - 59.07) ^b^ |
| Atherosclerosis | 0 | 0 (0 - 0) | 0 | 0 (0 - 0) | 0 | 0 (0 - 0) | 0 | 0 (0 - 0) | 0 | 0 (0 - 0) |
| Aortic Aneurysm and Dissection | 0 | 0 (0 - 246.96) | 1 | 14.2 (0.36 - 79.13) | 1 | 9.91 (0.25 - 55.22) | 2 | 16.59 (2.01 - 59.92) ^b^ | 4 | 13.04 (3.55 - 33.38) ^b^ |
| Other Diseases of Arteries, Arterioles, Capillaries | 0 | 0 (0 - 192.45) | 2 | 39.84 (4.82 - 143.9) ^b^ | 2 | 59.39 (7.19 - 214.53) ^b^ | 0 | 0 (0 - 0) | 4 | 38.82 (10.58 - 99.38) ^b^ |
| Pneumonia and Influenza | 1 | 35.97 (0.91 - 200.39) | 2 | 16.6 (2.01 - 59.96) ^b^ | 1 | 7.05 (0.18 - 39.26) | 4 | 22.46 (6.12 - 57.51) ^b^ | 8 | 17.08 (7.38 - 33.66) ^b^ |
| Chronic Obstructive Pulmonary Disease and Allied Cond | 2 | 12.95 (1.57 - 46.76) ^b^ | 11 | 17.67 (8.82 - 31.61) ^b^ | 15 | 20.51 (11.48 - 33.82) ^b^ | 14 | 38.64 (21.13 - 64.84) ^b^ | 42 | 22.45 (16.18 - 30.35) ^b^ |
| Stomach and Duodenal Ulcers | 0 | 0 (0 - 0) | 0 | 0 (0 - 0) | 0 | 0 (0 - 0) | 0 | 0 (0 - 0) | 0 | 0 (0 - 0) |
| Chronic Liver Disease and Cirrhosis | 5 | 89.13 (28.94 - 208) ^b^ | 7 | 42.43 (17.06 - 87.42) ^b^ | 4 | 37.9 (10.33 - 97.05) ^b^ | 1 | 31.08 (0.79 - 173.16) | 17 | 47.38 (27.6 - 75.86) ^b^ |
| Nephritis, Nephrotic Syndrome and Nephrosis | 1 | 45.27 (1.15 - 252.23) ^b^ | 1 | 13.8 (0.35 - 76.91) | 2 | 23.37 (2.83 - 84.42) ^b^ | 1 | 36.54 (0.93 - 203.58) | 5 | 24.1 (7.82 - 56.24) ^b^ |
| Complications of Pregnancy, Childbirth, Puerperium | 0 | 0 (0 - 4067.39) | 1 | 457.03 (11.57 - 2546.39) ^b^ | 0 | 0 (0 - 0) | 0 | 0 (0 - 0) | 1 | 323.1 (8.18 - 1800.21) ^b^ |
| Congenital Anomalies | 0 | 0 (0 - 0) | 0 | 0 (0 - 0) | 0 | 0 (0 - 0) | 0 | 0 (0 - 0) | 0 | 0 (0 - 0) |
| Certain Conditions Originating in Perinatal Period | 0 | 0 (0 - 0) | 0 | 0 (0 - 0) | 0 | 0 (0 - 0) | 0 | 0 (0 - 0) | 0 | 0 (0 - 0) |
| Symptoms, Signs, and Ill-Defined Conditions | 2 | 54.58 (6.61 - 197.15) ^b^ | 5 | 38.07 (12.36 - 88.85) ^b^ | 6 | 147 (53.95 - 319.97) ^b^ | 0 | 0 (0 - 0) | 13 | 62.27 (33.15 - 106.48) ^b^ |
| Accidents and Adverse Effects | 13 | 56.44 (30.05 - 96.52) ^b^ | 29 | 43.64 (29.23 - 62.68) ^b^ | 14 | 22.27 (12.18 - 37.37) ^b^ | 16 | 52.5 (30.01 - 85.26) ^b^ | 72 | 39.38 (30.82 - 49.6) ^b^ |
| Suicide and Self-Inflicted Injury | 3 | 104.32 (21.51 - 304.87) ^b^ | 6 | 77.17 (28.32 - 167.97) ^b^ | 3 | 136.44 (28.14 - 398.73) ^b^ | 0 | 0 (0 - 0) | 12 | 93.39 (48.26 - 163.13) ^b^ |
| Homicide and Legal Intervention | 0 | 0 (0 - 1514.21) | 1 | 242.85 (6.15 - 1353.06) ^b^ | 0 | 0 (0 - 0) | 0 | 0 (0 - 0) | 1 | 152.58 (3.86 - 850.12) ^b^ |
| Other Cause of Death | 17 | 64.28 (37.44 - 102.91) ^b^ | 23 | 25.81 (16.36 - 38.72) ^b^ | 25 | 28.86 (18.68 - 42.61) ^b^ | 18 | 55.14 (32.68 - 87.14) ^b^ | 83 | 35.34 (28.15 - 43.81) ^b^ |

**1** number of cancer patients who died due to each cause of death **2** 95% Confidence interval **b** P-value < 0.05

| **Supplementary Table 2.** Standardized-mortality ratios (SMRs) for each cause of death following laryngeal cancer diagnosis in patients aged 50 - 64 years. | | | | | | | | | | |
| --- | --- | --- | --- | --- | --- | --- | --- | --- | --- | --- |
| **Cause of death** | <1 year | | 1-5 years | | 5-10 years | | >10 years | | Total | |
|  | Observed^1^ | SMR (95% CI^2^) | Observed^1^ | SMR (95% CI^2^) | Observed^1^ | SMR (95% CI^2^) | Observed^1^ | SMR (95% CI^2^) | Observed^1^ | SMR (95% CI^2^) |
| All causes of death | 2,624 | 27.96 (26.9 - 29.05) ^b^ | 4,634 | 20.35 (19.77 - 20.95) ^b^ | 1,923 | 12.63 (12.07 - 13.21) ^b^ | 945 | 14.43 (13.52 - 15.38) ^b^ | 10,126 | 18.78 (18.41 - 19.15) ^b^ |
| Laryngeal cancer | 1,276 | 39.96 (37.8 - 42.21) ^b^ | 1,937 | 38.29 (36.61 - 40.04) ^b^ | 352 | 18.95 (17.02 - 21.04) ^b^ | 102 | 17.48 (14.26 - 21.22) ^b^ | 3,667 | 34.3 (33.19 - 35.42) ^b^ |
| Other non-laryngeal cancer causes of death | 760 | 23.09 (21.48 - 24.8) ^b^ | 1,641 | 19.33 (18.41 - 20.29) ^b^ | 745 | 13.22 (12.28 - 14.2) ^b^ | 333 | 15.91 (14.25 - 17.72) ^b^ | 3,479 | 17.83 (17.25 - 18.44) ^b^ |
| All non-cancer causes of death | 588 | 20.28 (18.67 - 21.98) ^b^ | 1,056 | 11.45 (10.77 - 12.16) ^b^ | 826 | 10.68 (9.97 - 11.44) ^b^ | 510 | 13.17 (12.05 - 14.36) ^b^ | 2,980 | 12.56 (12.11 - 13.02) ^b^ |
| In situ, benign or unknown behavior neoplasm | 8 | 16.11 (6.95 - 31.73) ^b^ | 23 | 14.47 (9.18 - 21.72) ^b^ | 11 | 10.06 (5.02 - 18) ^b^ | 10 | 9.29 (4.45 - 17.08) ^b^ | 52 | 12.22 (9.13 - 16.02) ^b^ |
| Tuberculosis | 1 | 48.12 (1.22 - 268.12) ^b^ | 1 | 13.79 (0.35 - 76.83) | 0 | 0 (0 - 0) | 0 | 0 (0 - 0) | 2 | 21.44 (2.6 - 77.43) ^b^ |
| Syphilis | 0 | 0 (0 - 0) | 0 | 0 (0 - 0) | 0 | 0 (0 - 0) | 0 | 0 (0 - 0) | 0 | 0 (0 - 0) |
| Septicemia | 29 | 38.82 (26 - 55.76) ^b^ | 24 | 11.31 (7.24 - 16.82) ^b^ | 19 | 9.34 (5.62 - 14.58) ^b^ | 12 | 39.03 (20.17 - 68.17) ^b^ | 84 | 16.12 (12.86 - 19.96) ^b^ |
| Other Infectious and Parasitic Diseases including HIV | 34 | 51.52 (35.68 - 71.99) ^b^ | 20 | 11.56 (7.06 - 17.85) ^b^ | 23 | 18.84 (11.94 - 28.27) ^b^ | 6 | 21.57 (7.92 - 46.95) ^b^ | 83 | 21.34 (17 - 26.45) ^b^ |
| Diabetes Mellitus | 16 | 17.91 (10.24 - 29.09) ^b^ | 21 | 6.88 (4.26 - 10.51) ^b^ | 28 | 10.17 (6.75 - 14.69) ^b^ | 16 | 11.99 (6.85 - 19.47) ^b^ | 81 | 10.08 (8.01 - 12.53) ^b^ |
| Alzheimer’s (ICD-9 and 10 only) | 1 | 6.52 (0.17 - 36.32) | 4 | 6.26 (1.71 - 16.04) ^b^ | 1 | 1.28 (0.03 - 7.11) | 5 | 6.12 (1.99 - 14.29) ^b^ | 11 | 4.6 (2.3 - 8.23) ^b^ |
| Diseases of Heart | 203 | 20.82 (18.05 - 23.89) ^b^ | 355 | 11.86 (10.65 - 13.16) ^b^ | 262 | 10.88 (9.6 - 12.28) ^b^ | 163 | 12.71 (10.83 - 14.82) ^b^ | 983 | 12.83 (12.04 - 13.66) ^b^ |
| Hypertension without Heart Disease | 5 | 16.58 (5.38 - 38.7) ^b^ | 12 | 14 (7.24 - 24.46) ^b^ | 8 | 12.78 (5.52 - 25.17) ^b^ | 5 | 18.65 (6.06 - 43.52) ^b^ | 30 | 14.61 (9.86 - 20.86) ^b^ |
| Cerebrovascular Diseases | 20 | 10.51 (6.42 - 16.23) ^b^ | 59 | 8.5 (6.47 - 10.96) ^b^ | 59 | 9.37 (7.13 - 12.09) ^b^ | 43 | 11.84 (8.57 - 15.95) ^b^ | 181 | 9.64 (8.29 - 11.15) ^b^ |
| Atherosclerosis | 1 | 29.19 (0.74 - 162.62) | 5 | 99.64 (32.35 - 232.54) ^b^ | 1 | 38.94 (0.99 - 216.97) | 0 | 0 (0 - 0) | 7 | 63.57 (25.56 - 130.97) ^b^ |
| Aortic Aneurysm and Dissection | 1 | 4.87 (0.12 - 27.13) | 7 | 9.93 (3.99 - 20.46) ^b^ | 6 | 10.29 (3.78 - 22.39) ^b^ | 4 | 17.77 (4.84 - 45.51) ^b^ | 18 | 10.47 (6.21 - 16.55) ^b^ |
| Other Diseases of Arteries, Arterioles, Capillaries | 7 | 37.49 (15.07 - 77.24) ^b^ | 7 | 17.97 (7.22 - 37.02) ^b^ | 5 | 13.38 (4.35 - 31.23) ^b^ | 2 | 84.73 (10.26 - 306.09) ^b^ | 21 | 21.57 (13.35 - 32.97) ^b^ |
| Pneumonia and Influenza | 14 | 14.65 (8.01 - 24.57) ^b^ | 36 | 12.12 (8.49 - 16.78) ^b^ | 23 | 8.59 (5.44 - 12.89) ^b^ | 18 | 15.94 (9.45 - 25.2) ^b^ | 91 | 11.77 (9.47 - 14.45) ^b^ |
| Chronic Obstructive Pulmonary Disease and Allied Cond | 64 | 15.76 (12.14 - 20.13) ^b^ | 136 | 9.43 (7.91 - 11.15) ^b^ | 138 | 10.65 (8.95 - 12.58) ^b^ | 91 | 15.82 (12.74 - 19.43) ^b^ | 429 | 11.53 (10.47 - 12.68) ^b^ |
| Stomach and Duodenal Ulcers | 2 | 33.07 (4.01 - 119.47) ^b^ | 4 | 24.24 (6.61 - 62.07) ^b^ | 3 | 42.43 (8.75 - 123.99) ^b^ | 0 | 0 (0 - 0) | 9 | 30.39 (13.89 - 57.68) ^b^ |
| Chronic Liver Disease and Cirrhosis | 20 | 27.6 (16.86 - 42.63) ^b^ | 31 | 13.21 (8.98 - 18.75) ^b^ | 23 | 12.99 (8.23 - 19.49) ^b^ | 9 | 26.54 (12.14 - 50.38) ^b^ | 83 | 16.02 (12.76 - 19.86) ^b^ |
| Nephritis, Nephrotic Syndrome and Nephrosis | 10 | 16.59 (7.96 - 30.51) ^b^ | 19 | 9.82 (5.91 - 15.33) ^b^ | 14 | 8.76 (4.79 - 14.7) ^b^ | 8 | 7.98 (3.44 - 15.72) ^b^ | 51 | 9.92 (7.39 - 13.05) ^b^ |
| Complications of Pregnancy, Childbirth, Puerperium | 0 | 0 (0 - 0) | 0 | 0 (0 - 0) | 0 | 0 (0 - 0) | 0 | 0 (0 - 0) | 0 | 0 (0 - 0) |
| Congenital Anomalies | 1 | 17.43 (0.44 - 97.12) | 2 | 12.57 (1.52 - 45.42) ^b^ | 1 | 9.26 (0.23 - 51.6) | 1 | 133.48 (3.38 - 743.72) ^b^ | 5 | 15.06 (4.89 - 35.15) ^b^ |
| Certain Conditions Originating in Perinatal Period | 0 | 0 (0 - 0) | 0 | 0 (0 - 0) | 0 | 0 (0 - 0) | 0 | 0 (0 - 0) | 0 | 0 (0 - 0) |
| Symptoms, Signs, and Ill-Defined Conditions | 20 | 34.58 (21.12 - 53.41) ^b^ | 26 | 17.62 (11.51 - 25.81) ^b^ | 15 | 14.78 (8.27 - 24.38) ^b^ | 3 | 14.88 (3.07 - 43.48) ^b^ | 64 | 19.57 (15.07 - 24.99) ^b^ |
| Accidents and Adverse Effects | 33 | 25.73 (17.71 - 36.13) ^b^ | 64 | 20.85 (16.05 - 26.62) ^b^ | 26 | 12.06 (7.88 - 17.67) ^b^ | 16 | 17.16 (9.81 - 27.87) ^b^ | 139 | 18.68 (15.7 - 22.06) ^b^ |
| Suicide and Self-Inflicted Injury | 13 | 21.76 (11.59 - 37.22) ^b^ | 33 | 19.37 (13.33 - 27.2) ^b^ | 16 | 16.95 (9.69 - 27.53) ^b^ | 5 | 13.41 (4.36 - 31.3) ^b^ | 67 | 18.52 (14.35 - 23.52) ^b^ |
| Homicide and Legal Intervention | 2 | 29.92 (3.62 - 108.1) ^b^ | 3 | 15.78 (3.25 - 46.12) ^b^ | 3 | 150.21 (30.98 - 438.99) ^b^ | 0 | 0 (0 - 0) | 8 | 28.89 (12.47 - 56.92) ^b^ |
| Other Cause of Death | 83 | 17.81 (14.18 - 22.08) ^b^ | 164 | 10.45 (8.91 - 12.18) ^b^ | 141 | 9.98 (8.4 - 11.77) ^b^ | 93 | 11.34 (9.15 - 13.89) ^b^ | 481 | 11.27 (10.28 - 12.32) ^b^ |

**1** number of cancer patients who died due to each cause of death **2** 95% Confidence interval **b** P-value < 0.05

| **Supplementary Table 3.** Standardized-mortality ratios (SMRs) for each cause of death following laryngeal cancer diagnosis in patients aged >64 years. | | | | | | | | | | |
| --- | --- | --- | --- | --- | --- | --- | --- | --- | --- | --- |
| **Cause of death** | <1 year | | 1-5 years | | 5-10 years | | >10 years | | Total | |
|  | Observed^1^ | SMR (95% CI2) | Observed^1^ | SMR (95% CI2) | Observed^1^ | SMR (95% CI2) | Observed^1^ | SMR (95% CI2) | Observed^1^ | SMR (95% CI2) |
| All causes of death | 4,114 | 7.75 (7.51 - 7.99) ^b^ | 5,658 | 4.4 (4.29 - 4.52) ^b^ | 2,636 | 3.42 (3.29 - 3.56) ^b^ | 1,108 | 4.36 (4.11 - 4.62) ^b^ | 13,516 | 4.76 (4.68 - 4.84) ^b^ |
| Laryngeal cancer | 2,012 | 14.18 (13.56 - 14.81) ^b^ | 1,941 | 10.21 (9.77 - 10.68) ^b^ | 306 | 4.74 (4.22 - 5.3) ^b^ | 76 | 4.28 (3.37 - 5.36) ^b^ | 4,335 | 10.46 (10.15 - 10.78) ^b^ |
| Other non-laryngeal cancer causes of death | 975 | 7.45 (6.99 - 7.93) ^b^ | 1,652 | 5.25 (5 - 5.5) ^b^ | 753 | 4.06 (3.78 - 4.36) ^b^ | 306 | 5.17 (4.61 - 5.79) ^b^ | 3,686 | 5.34 (5.17 - 5.52) ^b^ |
| All non-cancer causes of death | 1,127 | 4.36 (4.11 - 4.62) ^b^ | 2,065 | 2.64 (2.53 - 2.76) ^b^ | 1,577 | 3.03 (2.88 - 3.19) ^b^ | 726 | 4.09 (3.8 - 4.4) ^b^ | 5,495 | 3.16 (3.08 - 3.25) ^b^ |
| In situ, benign or unknown behavior neoplasm | 13 | 4.52 (2.41 - 7.73) ^b^ | 31 | 4.17 (2.84 - 5.92) ^b^ | 12 | 2.63 (1.36 - 4.6) ^b^ | 8 | 3.72 (1.61 - 7.33) ^b^ | 64 | 3.76 (2.9 - 4.8) ^b^ |
| Tuberculosis | 0 | 0 (0 - 207.91) | 1 | 30.58 (0.77 - 170.36) | 0 | 0 (0 - 0) | 0 | 0 (0 - 0) | 1 | 19.82 (0.5 - 110.44) |
| Syphilis | 0 | 0 (0 - 0) | 0 | 0 (0 - 0) | 0 | 0 (0 - 0) | 0 | 0 (0 - 0) | 0 | 0 (0 - 0) |
| Septicemia | 36 | 7.84 (5.49 - 10.85) ^b^ | 48 | 4.75 (3.5 - 6.29) ^b^ | 23 | 3.45 (2.19 - 5.18) ^b^ | 14 | 4.38 (2.39 - 7.35) ^b^ | 121 | 4.93 (4.09 - 5.89) ^b^ |
| Other Infectious and Parasitic Diseases including HIV | 15 | 7.33 (4.1 - 12.1) ^b^ | 24 | 5.28 (3.39 - 7.86) ^b^ | 9 | 3.3 (1.51 - 6.27) ^b^ | 5 | 4.07 (1.32 - 9.49) ^b^ | 53 | 5.03 (3.77 - 6.58) ^b^ |
| Diabetes Mellitus | 22 | 4.14 (2.59 - 6.26) ^b^ | 56 | 3.18 (2.4 - 4.13) ^b^ | 38 | 2.53 (1.79 - 3.47) ^b^ | 26 | 4.85 (3.17 - 7.1) ^b^ | 142 | 3.28 (2.76 - 3.86) ^b^ |
| Alzheimer’s (ICD-9 and 10 only) | 8 | 0.86 (0.37 - 1.7) | 42 | 1.22 (0.88 - 1.65) | 57 | 2.33 (1.76 - 3.02) ^b^ | 27 | 3.5 (2.31 - 5.09) ^b^ | 134 | 1.77 (1.48 - 2.09) ^b^ |
| Diseases of Heart | 418 | 4.64 (4.2 - 5.1) ^b^ | 695 | 2.63 (2.44 - 2.84) ^b^ | 495 | 2.85 (2.61 - 3.12) ^b^ | 232 | 3.9 (3.41 - 4.44) ^b^ | 1,840 | 3.13 (2.99 - 3.28) ^b^ |
| Hypertension without Heart Disease | 17 | 4.33 (2.52 - 6.93) ^b^ | 26 | 2.34 (1.53 - 3.43) ^b^ | 23 | 2.5 (1.59 - 3.75) ^b^ | 9 | 3.84 (1.76 - 7.3) ^b^ | 75 | 2.82 (2.22 - 3.54) ^b^ |
| Cerebrovascular Diseases | 48 | 2.87 (2.11 - 3.8) ^b^ | 147 | 2.93 (2.48 - 3.45) ^b^ | 109 | 3.36 (2.76 - 4.05) ^b^ | 41 | 4.52 (3.24 - 6.13) ^b^ | 345 | 3.18 (2.85 - 3.54) ^b^ |
| Atherosclerosis | 6 | 5.9 (2.16 - 12.84) ^b^ | 6 | 2.12 (0.78 - 4.61) | 9 | 7.64 (3.49 - 14.5) ^b^ | 0 | 0 (0 - 0) | 21 | 4.17 (2.58 - 6.38) ^b^ |
| Aortic Aneurysm and Dissection | 8 | 4.7 (2.03 - 9.26) ^b^ | 21 | 3.91 (2.42 - 5.98) ^b^ | 15 | 4.23 (2.37 - 6.97) ^b^ | 4 | 5.04 (1.37 - 12.91) ^b^ | 48 | 4.21 (3.1 - 5.58) ^b^ |
| Other Diseases of Arteries, Arterioles, Capillaries | 10 | 7.99 (3.83 - 14.7) ^b^ | 15 | 4.77 (2.67 - 7.87) ^b^ | 8 | 4.49 (1.94 - 8.85) ^b^ | 2 | 10.47 (1.27 - 37.81) ^b^ | 35 | 5.5 (3.83 - 7.64) ^b^ |
| Pneumonia and Influenza | 51 | 4.4 (3.28 - 5.78) ^b^ | 81 | 2.45 (1.95 - 3.04) ^b^ | 59 | 3.15 (2.4 - 4.07) ^b^ | 32 | 4.76 (3.26 - 6.72) ^b^ | 223 | 3.18 (2.78 - 3.63) ^b^ |
| Chronic Obstructive Pulmonary Disease and Allied Cond | 187 | 4.98 (4.29 - 5.75) ^b^ | 366 | 3.19 (2.87 - 3.53) ^b^ | 276 | 3.78 (3.35 - 4.25) ^b^ | 120 | 4.72 (3.91 - 5.64) ^b^ | 949 | 3.78 (3.55 - 4.03) ^b^ |
| Stomach and Duodenal Ulcers | 5 | 6.45 (2.09 - 15.06) ^b^ | 8 | 3.25 (1.4 - 6.4) ^b^ | 3 | 2.07 (0.43 - 6.05) | 2 | 4.65 (0.56 - 16.78) | 18 | 3.52 (2.08 - 5.56) ^b^ |
| Chronic Liver Disease and Cirrhosis | 8 | 6.54 (2.82 - 12.89) ^b^ | 25 | 8.34 (5.4 - 12.31) ^b^ | 9 | 5.41 (2.47 - 10.27) ^b^ | 2 | 6.01 (0.73 - 21.7) | 44 | 7.08 (5.14 - 9.5) ^b^ |
| Nephritis, Nephrotic Syndrome and Nephrosis | 15 | 2.04 (1.14 - 3.37) ^b^ | 45 | 1.82 (1.33 - 2.43) ^b^ | 42 | 2.8 (2.02 - 3.79) ^b^ | 15 | 4.13 (2.31 - 6.8) ^b^ | 117 | 2.31 (1.91 - 2.76) ^b^ |
| Complications of Pregnancy, Childbirth, Puerperium | 0 | 0 (0 - 0) | 0 | 0 (0 - 0) | 0 | 0 (0 - 0) | 0 | 0 (0 - 0) | 0 | 0 (0 - 0) |
| Congenital Anomalies | 0 | 0 (0 - 25.13) | 1 | 1.84 (0.05 - 10.26) | 1 | 2.47 (0.06 - 13.78) | 0 | 0 (0 - 0) | 2 | 1.83 (0.22 - 6.6) |
| Certain Conditions Originating in Perinatal Period | 0 | 0 (0 - 0) | 0 | 0 (0 - 0) | 0 | 0 (0 - 0) | 0 | 0 (0 - 0) | 0 | 0 (0 - 0) |
| Symptoms, Signs, and Ill-Defined Conditions | 13 | 3.35 (1.78 - 5.72) ^b^ | 20 | 1.38 (0.84 - 2.13) | 30 | 2.11 (1.43 - 3.02) ^b^ | 17 | 4.42 (2.58 - 7.08) ^b^ | 80 | 2.2 (1.74 - 2.74) ^b^ |
| Accidents and Adverse Effects | 29 | 3.66 (2.45 - 5.26) ^b^ | 56 | 2.27 (1.71 - 2.95) ^b^ | 59 | 4.37 (3.33 - 5.64) ^b^ | 16 | 5.19 (2.96 - 8.42) ^b^ | 160 | 3.25 (2.77 - 3.8) ^b^ |
| Suicide and Self-Inflicted Injury | 26 | 11.27 (7.36 - 16.51) ^b^ | 24 | 4.82 (3.09 - 7.17) ^b^ | 13 | 6.81 (3.62 - 11.64) ^b^ | 2 | 3.78 (0.46 - 13.64) | 65 | 6.68 (5.16 - 8.52) ^b^ |
| Homicide and Legal Intervention | 2 | 7.2 (0.87 - 26.02) | 7 | 15.06 (6.06 - 31.03) ^b^ | 0 | 0 (0 - 18.1) | 1 | 2.22 (0.06 - 12.39) | 10 | 7.16 (3.44 - 13.18) ^b^ |
| Other Cause of Death | 190 | 4.09 (3.53 - 4.72) ^b^ | 320 | 2.17 (1.94 - 2.43) ^b^ | 287 | 2.73 (2.43 - 3.07) ^b^ | 151 | 3.65 (3.09 - 4.28) ^b^ | 948 | 2.79 (2.61 - 2.97) ^b^ |

**1** number of cancer patients who died due to each cause of death **2** 95% Confidence interval **b** P-value < 0.05

| **Supplementary Table 4.** Standardized-mortality ratios (SMRs) for each cause of death following laryngeal cancer diagnosis in male patients. | | | | | | | | | | |
| --- | --- | --- | --- | --- | --- | --- | --- | --- | --- | --- |
| **Cause of death** | <1 year | | 1-5 years | | 5-10 years | | >10 years | | Total | |
|  | Observed^1^ | SMR (95% CI^2^) | Observed^1^ | SMR (95% CI^2^) | Observed^1^ | SMR (95% CI^2^) | Observed^1^ | SMR (95% CI^2^) | Observed^1^ | SMR (95% CI^2^) |
| All causes of death | 5,740 | 10.65 (10.38 - 10.93) ^b^ | 8,946 | 6.83 (6.69 - 6.98) ^b^ | 3,873 | 4.85 (4.7 - 5) ^b^ | 1,859 | 6.58 (6.29 - 6.89) ^b^ | 20,418 | 6.97 (6.88 - 7.07) ^b^ |
| Laryngeal cancer | 2,807 | 18.86 (18.17 - 19.57) ^b^ | 3,419 | 16.49 (15.94 - 17.05) ^b^ | 566 | 7.86 (7.22 - 8.53) ^b^ | 175 | 8.39 (7.19 - 9.73) ^b^ | 6,967 | 15.52 (15.15 - 15.88) ^b^ |
| Other non-laryngeal cancer causes of death | 1,483 | 10.41 (9.89 - 10.96) ^b^ | 2,889 | 8.26 (7.96 - 8.57) ^b^ | 1,272 | 6.04 (5.71 - 6.38) ^b^ | 591 | 8.16 (7.52 - 8.85) ^b^ | 6,235 | 8.04 (7.84 - 8.25) ^b^ |
| All non-cancer causes of death | 1,450 | 5.86 (5.56 - 6.17) ^b^ | 2,638 | 3.51 (3.37 - 3.64) ^b^ | 2,035 | 3.94 (3.77 - 4.12) ^b^ | 1,093 | 5.78 (5.44 - 6.13) ^b^ | 7,216 | 4.23 (4.14 - 4.33) ^b^ |
| In situ, benign or unknown behavior neoplasm | 18 | 6.26 (3.71 - 9.9) ^b^ | 42 | 5.55 (4 - 7.5) ^b^ | 20 | 3.89 (2.38 - 6.01) ^b^ | 16 | 5.06 (2.89 - 8.22) ^b^ | 96 | 5.12 (4.15 - 6.26) ^b^ |
| Tuberculosis | 1 | 25.96 (0.66 - 144.63) | 2 | 19.01 (2.3 - 68.66) ^b^ | 0 | 0 (0 - 0) | 0 | 0 (0 - 0) | 3 | 20.87 (4.3 - 60.99) ^b^ |
| Syphilis | 0 | 0 (0 - 0) | 0 | 0 (0 - 0) | 0 | 0 (0 - 0) | 0 | 0 (0 - 0) | 0 | 0 (0 - 0) |
| Septicemia | 51 | 11.12 (8.28 - 14.62) ^b^ | 56 | 5.19 (3.92 - 6.74) ^b^ | 34 | 3.99 (2.76 - 5.58) ^b^ | 27 | 7.81 (5.14 - 11.36) ^b^ | 168 | 6.14 (5.25 - 7.15) ^b^ |
| Other Infectious and Parasitic Diseases including HIV | 42 | 16.81 (12.12 - 22.73) ^b^ | 42 | 7.1 (5.12 - 9.6) ^b^ | 31 | 8 (5.44 - 11.36) ^b^ | 13 | 8.27 (4.41 - 14.15) ^b^ | 128 | 9.24 (7.71 - 10.98) ^b^ |
| Diabetes Mellitus | 30 | 5.38 (3.63 - 7.68) ^b^ | 66 | 3.59 (2.78 - 4.57) ^b^ | 59 | 3.74 (2.85 - 4.83) ^b^ | 38 | 7.46 (5.28 - 10.24) ^b^ | 193 | 4.31 (3.72 - 4.96) ^b^ |
| Alzheimer’s (ICD-9 and 10 only) | 9 | 1.19 (0.55 - 2.26) | 36 | 1.32 (0.92 - 1.82) | 46 | 2.38 (1.74 - 3.17) ^b^ | 27 | 3.56 (2.35 - 5.18) ^b^ | 118 | 1.91 (1.58 - 2.29) ^b^ |
| Diseases of Heart | 531 | 6.13 (5.62 - 6.68) ^b^ | 923 | 3.62 (3.39 - 3.86) ^b^ | 654 | 3.8 (3.52 - 4.1) ^b^ | 357 | 5.49 (4.93 - 6.09) ^b^ | 2,465 | 4.26 (4.09 - 4.43) ^b^ |
| Hypertension without Heart Disease | 18 | 4.72 (2.8 - 7.47) ^b^ | 32 | 2.9 (1.98 - 4.09) ^b^ | 29 | 3.1 (2.08 - 4.45) ^b^ | 13 | 5.02 (2.67 - 8.59) ^b^ | 92 | 3.43 (2.77 - 4.21) ^b^ |
| Cerebrovascular Diseases | 54 | 3.41 (2.56 - 4.45) ^b^ | 174 | 3.57 (3.06 - 4.14) ^b^ | 137 | 4.15 (3.49 - 4.91) ^b^ | 72 | 6.47 (5.06 - 8.14) ^b^ | 437 | 4.02 (3.65 - 4.41) ^b^ |
| Atherosclerosis | 6 | 6.45 (2.37 - 14.04) ^b^ | 8 | 3.46 (1.5 - 6.82) ^b^ | 6 | 6.84 (2.51 - 14.88) ^b^ | 0 | 0 (0 - 0) | 20 | 4.86 (2.97 - 7.5) ^b^ |
| Aortic Aneurysm and Dissection | 8 | 5.2 (2.25 - 10.25) ^b^ | 23 | 4.51 (2.86 - 6.76) ^b^ | 17 | 4.89 (2.85 - 7.83) ^b^ | 8 | 10.3 (4.45 - 20.3) ^b^ | 56 | 5.14 (3.88 - 6.68) ^b^ |
| Other Diseases of Arteries, Arterioles, Capillaries | 15 | 12.08 (6.76 - 19.92) ^b^ | 19 | 6.89 (4.15 - 10.77) ^b^ | 8 | 5.04 (2.17 - 9.93) ^b^ | 3 | 23.95 (4.94 - 69.99) ^b^ | 45 | 7.88 (5.75 - 10.54) ^b^ |
| Pneumonia and Influenza | 59 | 5.31 (4.05 - 6.86) ^b^ | 91 | 2.81 (2.26 - 3.45) ^b^ | 74 | 3.86 (3.03 - 4.85) ^b^ | 46 | 6.34 (4.64 - 8.45) ^b^ | 270 | 3.86 (3.42 - 4.35) ^b^ |
| Chronic Obstructive Pulmonary Disease and Allied Cond | 182 | 5.34 (4.59 - 6.18) ^b^ | 371 | 3.53 (3.18 - 3.91) ^b^ | 304 | 4.45 (3.96 - 4.98) ^b^ | 171 | 6.92 (5.92 - 8.04) ^b^ | 1,028 | 4.43 (4.16 - 4.71) ^b^ |
| Stomach and Duodenal Ulcers | 6 | 7.74 (2.84 - 16.85) ^b^ | 8 | 3.18 (1.37 - 6.27) ^b^ | 4 | 2.73 (0.74 - 6.98) | 2 | 4.65 (0.56 - 16.78) | 20 | 3.86 (2.36 - 5.96) ^b^ |
| Chronic Liver Disease and Cirrhosis | 26 | 13.89 (9.08 - 20.36) ^b^ | 58 | 11.26 (8.55 - 14.56) ^b^ | 31 | 9.17 (6.23 - 13.02) ^b^ | 12 | 17.04 (8.81 - 29.77) ^b^ | 127 | 11.44 (9.53 - 13.61) ^b^ |
| Nephritis, Nephrotic Syndrome and Nephrosis | 21 | 3.02 (1.87 - 4.62) ^b^ | 53 | 2.25 (1.69 - 2.95) ^b^ | 53 | 3.65 (2.73 - 4.77) ^b^ | 16 | 3.98 (2.27 - 6.46) ^b^ | 143 | 2.92 (2.46 - 3.44) ^b^ |
| Complications of Pregnancy, Childbirth, Puerperium | 0 | 0 (0 - 0) | 0 | 0 (0 - 0) | 0 | 0 (0 - 0) | 0 | 0 (0 - 0) | 0 | 0 (0 - 0) |
| Congenital Anomalies | 0 | 0 (0 - 19.74) | 2 | 2.92 (0.35 - 10.56) | 2 | 3.9 (0.47 - 14.11) | 1 | 133.48 (3.38 - 743.72) ^b^ | 5 | 3.59 (1.17 - 8.39) ^b^ |
| Certain Conditions Originating in Perinatal Period | 0 | 0 (0 - 0) | 0 | 0 (0 - 0) | 0 | 0 (0 - 0) | 0 | 0 (0 - 0) | 0 | 0 (0 - 0) |
| Symptoms, Signs, and Ill-Defined Conditions | 32 | 8.07 (5.52 - 11.39) ^b^ | 38 | 2.76 (1.96 - 3.79) ^b^ | 42 | 3.22 (2.32 - 4.35) ^b^ | 17 | 5.17 (3.01 - 8.27) ^b^ | 129 | 3.79 (3.16 - 4.5) ^b^ |
| Accidents and Adverse Effects | 64 | 7.85 (6.04 - 10.02) ^b^ | 117 | 4.71 (3.9 - 5.65) ^b^ | 77 | 5.26 (4.15 - 6.58) ^b^ | 39 | 9.92 (7.05 - 13.55) ^b^ | 297 | 5.76 (5.12 - 6.46) ^b^ |
| Suicide and Self-Inflicted Injury | 41 | 14.11 (10.13 - 19.15) ^b^ | 61 | 9.08 (6.95 - 11.66) ^b^ | 32 | 11.38 (7.79 - 16.07) ^b^ | 6 | 6.91 (2.54 - 15.04) ^b^ | 140 | 10.52 (8.85 - 12.42) ^b^ |
| Homicide and Legal Intervention | 3 | 10.28 (2.12 - 30.04) ^b^ | 9 | 17.42 (7.96 - 33.07) ^b^ | 2 | 8.98 (1.09 - 32.44) ^b^ | 1 | 2.22 (0.06 - 12.39) | 15 | 10.13 (5.67 - 16.71) ^b^ |
| Other Cause of Death | 233 | 5.27 (4.61 - 5.99) ^b^ | 407 | 2.87 (2.6 - 3.17) ^b^ | 373 | 3.55 (3.2 - 3.93) ^b^ | 208 | 4.85 (4.21 - 5.56) ^b^ | 1,221 | 3.66 (3.46 - 3.87) ^b^ |

**1** number of cancer patients who died due to each cause of death **2** 95% Confidence interval **b** P-value < 0.05

| **Supplementary Table 5.** Standardized-mortality ratios (SMRs) for each cause of death following laryngeal cancer diagnosis in female patients. | | | | | | | | | | |
| --- | --- | --- | --- | --- | --- | --- | --- | --- | --- | --- |
| **Cause of death** | <1 year | | 1-5 years | | 5-10 years | | >10 years | | Total | |
|  | Observed^1^ | SMR (95% CI^2^) | Observed^1^ | SMR (95% CI^2^) | Observed^1^ | SMR (95% CI^2^) | Observed^1^ | SMR (95% CI^2^) | Observed^1^ | SMR (95% CI^2^) |
| All causes of death | 1,425 | 15.41 (14.62 - 16.23) ^b^ | 2,175 | 9.92 (9.51 - 10.35) ^b^ | 983 | 7.31 (6.86 - 7.79) ^b^ | 406 | 9.44 (8.54 - 10.4) ^b^ | 4,989 | 10.2 (9.92 - 10.49) ^b^ |
| Laryngeal cancer | 682 | 24.77 (22.94 - 26.7) ^b^ | 857 | 22.91 (21.41 - 24.5) ^b^ | 157 | 11.74 (9.97 - 13.72) ^b^ | 42 | 11.51 (8.29 - 15.55) ^b^ | 1,738 | 21.2 (20.22 - 22.23) ^b^ |
| Other non-laryngeal cancer causes of death | 394 | 16.79 (15.17 - 18.53) ^b^ | 674 | 12.2 (11.3 - 13.16) ^b^ | 333 | 9.55 (8.55 - 10.63) ^b^ | 121 | 12.22 (10.14 - 14.6) ^b^ | 1,522 | 12.33 (11.71 - 12.96) ^b^ |
| All non-cancer causes of death | 349 | 8.42 (7.56 - 9.35) ^b^ | 644 | 5.09 (4.7 - 5.5) ^b^ | 493 | 5.72 (5.23 - 6.25) ^b^ | 243 | 8.25 (7.24 - 9.35) ^b^ | 1,729 | 6.1 (5.81 - 6.39) ^b^ |
| In situ, benign or unknown behavior neoplasm | 6 | 11.54 (4.24 - 25.12) ^b^ | 15 | 9.97 (5.58 - 16.44) ^b^ | 4 | 7.22 (1.97 - 18.49) ^b^ | 3 | 35.34 (7.29 - 103.27) ^b^ | 28 | 10.51 (6.99 - 15.2) ^b^ |
| Tuberculosis | 0 | 0 (0 - 0) | 0 | 0 (0 - 0) | 0 | 0 (0 - 0) | 0 | 0 (0 - 0) | 0 | 0 (0 - 0) |
| Syphilis | 0 | 0 (0 - 0) | 0 | 0 (0 - 0) | 0 | 0 (0 - 0) | 0 | 0 (0 - 0) | 0 | 0 (0 - 0) |
| Septicemia | 19 | 22.94 (13.81 - 35.83) ^b^ | 21 | 12.46 (7.71 - 19.04) ^b^ | 11 | 28.04 (14 - 50.16) ^b^ | 2 | 17.66 (2.14 - 63.78) ^b^ | 53 | 17.55 (13.15 - 22.96) ^b^ |
| Other Infectious and Parasitic Diseases including HIV | 14 | 45.79 (25.03 - 76.82) ^b^ | 11 | 16.97 (8.47 - 30.36) ^b^ | 7 | 25.65 (10.31 - 52.85) ^b^ | 1 | 522.44 (13.23 - 2910.87) ^b^ | 33 | 26.85 (18.48 - 37.71) ^b^ |
| Diabetes Mellitus | 8 | 12.37 (5.34 - 24.37) ^b^ | 12 | 5.18 (2.67 - 9.04) ^b^ | 8 | 3.87 (1.67 - 7.63) ^b^ | 6 | 3.71 (1.36 - 8.08) ^b^ | 34 | 5.11 (3.54 - 7.15) ^b^ |
| Alzheimer’s (ICD-9 and 10 only) | 0 | 0 (0 - 1.95) | 10 | 1.31 (0.63 - 2.41) | 12 | 2.03 (1.05 - 3.54) ^b^ | 5 | 5.29 (1.72 - 12.33) ^b^ | 27 | 1.65 (1.08 - 2.4) ^b^ |
| Diseases of Heart | 113 | 8.18 (6.74 - 9.83) ^b^ | 169 | 4.21 (3.6 - 4.89) ^b^ | 138 | 5.13 (4.31 - 6.06) ^b^ | 68 | 8.36 (6.49 - 10.59) ^b^ | 488 | 5.48 (5 - 5.99) ^b^ |
| Hypertension without Heart Disease | 6 | 14.01 (5.14 - 30.5) ^b^ | 7 | 7.37 (2.96 - 15.18) ^b^ | 2 | 4.09 (0.5 - 14.78) | 2 | 47.93 (5.8 - 173.13) ^b^ | 17 | 8.9 (5.19 - 14.26) ^b^ |
| Cerebrovascular Diseases | 14 | 4.84 (2.65 - 8.12) ^b^ | 43 | 4.99 (3.61 - 6.72) ^b^ | 37 | 6.29 (4.43 - 8.67) ^b^ | 16 | 9.8 (5.6 - 15.91) ^b^ | 110 | 5.78 (4.75 - 6.97) ^b^ |
| Atherosclerosis | 1 | 8.22 (0.21 - 45.83) | 3 | 5.22 (1.08 - 15.26) ^b^ | 4 | 12.26 (3.34 - 31.39) ^b^ | 0 | 0 (0 - 0) | 8 | 7.82 (3.38 - 15.42) ^b^ |
| Aortic Aneurysm and Dissection | 1 | 2.6 (0.07 - 14.49) | 6 | 5.75 (2.11 - 12.52) ^b^ | 5 | 6.62 (2.15 - 15.44) ^b^ | 2 | 5.52 (0.67 - 19.94) | 14 | 5.5 (3.01 - 9.23) ^b^ |
| Other Diseases of Arteries, Arterioles, Capillaries | 2 | 9.29 (1.13 - 33.57) ^b^ | 5 | 6.04 (1.96 - 14.1) ^b^ | 7 | 11.64 (4.68 - 23.99) ^b^ | 1 | 11.18 (0.28 - 62.31) | 15 | 8.65 (4.84 - 14.27) ^b^ |
| Pneumonia and Influenza | 7 | 4.75 (1.91 - 9.78) ^b^ | 28 | 7.41 (4.92 - 10.71) ^b^ | 9 | 3.78 (1.73 - 7.17) ^b^ | 8 | 10.34 (4.47 - 20.38) ^b^ | 52 | 6.18 (4.62 - 8.11) ^b^ |
| Chronic Obstructive Pulmonary Disease and Allied Cond | 71 | 9.22 (7.2 - 11.64) ^b^ | 142 | 5.72 (4.82 - 6.74) ^b^ | 125 | 6.81 (5.67 - 8.11) ^b^ | 54 | 7.92 (5.95 - 10.34) ^b^ | 392 | 6.8 (6.14 - 7.5) ^b^ |
| Stomach and Duodenal Ulcers | 1 | 16.58 (0.42 - 92.38) | 4 | 34.81 (9.49 - 89.14) ^b^ | 2 | 36.96 (4.48 - 133.51) ^b^ | 0 | 0 (0 - 0) | 7 | 30.52 (12.27 - 62.89) ^b^ |
| Chronic Liver Disease and Cirrhosis | 7 | 52.78 (21.22 - 108.75) ^b^ | 5 | 13.94 (4.52 - 32.52) ^b^ | 5 | 31.19 (10.13 - 72.78) ^b^ | 0 | 0 (0 - 0) | 17 | 26.08 (15.19 - 41.76) ^b^ |
| Nephritis, Nephrotic Syndrome and Nephrosis | 5 | 4.91 (1.59 - 11.46) ^b^ | 12 | 3.69 (1.91 - 6.45) ^b^ | 5 | 2.33 (0.76 - 5.44) | 8 | 12.46 (5.38 - 24.56) ^b^ | 30 | 4.25 (2.87 - 6.07) ^b^ |
| Complications of Pregnancy, Childbirth, Puerperium | 0 | 0 (0 - 4067.39) | 1 | 457.03 (11.57 - 2546.39) ^b^ | 0 | 0 (0 - 0) | 0 | 0 (0 - 0) | 1 | 323.1 (8.18 - 1800.21) ^b^ |
| Congenital Anomalies | 1 | 57.85 (1.46 - 322.34) ^b^ | 1 | 55.89 (1.41 - 311.38) ^b^ | 0 | 0 (0 - 0) | 0 | 0 (0 - 0) | 2 | 56.85 (6.89 - 205.37) ^b^ |
| Certain Conditions Originating in Perinatal Period | 0 | 0 (0 - 0) | 0 | 0 (0 - 0) | 0 | 0 (0 - 0) | 0 | 0 (0 - 0) | 0 | 0 (0 - 0) |
| Symptoms, Signs, and Ill-Defined Conditions | 3 | 5.6 (1.15 - 16.37) ^b^ | 13 | 5.59 (2.98 - 9.56) ^b^ | 9 | 4.08 (1.87 - 7.75) ^b^ | 3 | 3.97 (0.82 - 11.61) | 28 | 4.81 (3.2 - 6.95) ^b^ |
| Accidents and Adverse Effects | 11 | 8.62 (4.3 - 15.43) ^b^ | 32 | 8.91 (6.09 - 12.58) ^b^ | 22 | 13.35 (8.36 - 20.21) ^b^ | 9 | 23.18 (10.6 - 44.01) ^b^ | 74 | 10.72 (8.42 - 13.46) ^b^ |
| Suicide and Self-Inflicted Injury | 1 | 34.96 (0.89 - 194.8) | 2 | 46.87 (5.68 - 169.3) ^b^ | 0 | 0 (0 - 57.57) | 1 | 29.28 (0.74 - 163.15) | 4 | 23.6 (6.43 - 60.42) ^b^ |
| Homicide and Legal Intervention | 1 | 18.16 (0.46 - 101.2) | 2 | 14.06 (1.7 - 50.78) ^b^ | 1 | 974.32 (24.67 - 5428.56) ^b^ | 0 | 0 (0 - 0) | 4 | 20.16 (5.49 - 51.63) ^b^ |
| Other Cause of Death | 57 | 8.02 (6.07 - 10.39) ^b^ | 100 | 4.52 (3.68 - 5.5) ^b^ | 80 | 5.34 (4.24 - 6.65) ^b^ | 54 | 7.69 (5.78 - 10.04) ^b^ | 291 | 5.68 (5.05 - 6.38) ^b^ |

**1** number of cancer patients who died due to each cause of death **2** 95% Confidence interval **b** P-value < 0.05

| **Supplementary Table 6.** Standardized-mortality ratios (SMRs) for each cause of death following laryngeal cancer diagnosis in White patients. | | | | | | | | | | |
| --- | --- | --- | --- | --- | --- | --- | --- | --- | --- | --- |
| **Cause of death** | <1 year | | 1-5 years | | 5-10 years | | >10 years | | Total | |
|  | Observed^1^ | SMR (95% CI^2^) | Observed^1^ | SMR (95% CI^2^) | Observed^1^ | SMR (95% CI^2^) | Observed^1^ | SMR (95% CI^2^) | Observed^1^ | SMR (95% CI^2^) |
| All causes of death | 5,559 | 10.72 (10.44 - 11) ^b^ | 8,860 | 6.92 (6.77 - 7.06) ^b^ | 4,032 | 5.07 (4.92 - 5.23) ^b^ | 1,885 | 6.77 (6.47 - 7.09) ^b^ | 20,336 | 7.08 (6.98 - 7.18) ^b^ |
| Laryngeal cancer | 2,749 | 19.75 (19.02 - 20.51) ^b^ | 3,305 | 17.07 (16.49 - 17.66) ^b^ | 586 | 8.35 (7.69 - 9.05) ^b^ | 166 | 8.32 (7.1 - 9.68) ^b^ | 6,806 | 16.09 (15.71 - 16.48) ^b^ |
| Other non-laryngeal cancer causes of death | 1,412 | 10.66 (10.11 - 11.23) ^b^ | 2,812 | 8.54 (8.23 - 8.86) ^b^ | 1,318 | 6.49 (6.15 - 6.85) ^b^ | 578 | 8.56 (7.87 - 9.28) ^b^ | 6,120 | 8.36 (8.15 - 8.57) ^b^ |
| All non-cancer causes of death | 1,398 | 5.66 (5.36 - 5.96) ^b^ | 2,743 | 3.62 (3.48 - 3.76) ^b^ | 2,128 | 4.08 (3.91 - 4.26) ^b^ | 1,141 | 5.98 (5.64 - 6.34) ^b^ | 7,410 | 4.31 (4.22 - 4.41) ^b^ |
| In situ, benign or unknown behavior neoplasm | 17 | 5.88 (3.43 - 9.42) ^b^ | 44 | 5.56 (4.04 - 7.46) ^b^ | 20 | 3.72 (2.27 - 5.75) ^b^ | 19 | 5.85 (3.52 - 9.14) ^b^ | 100 | 5.15 (4.19 - 6.26) ^b^ |
| Tuberculosis | 1 | 56.36 (1.43 - 314.03) ^b^ | 1 | 30.58 (0.77 - 170.36) | 0 | 0 (0 - 0) | 0 | 0 (0 - 0) | 2 | 39.65 (4.8 - 143.21) ^b^ |
| Syphilis | 0 | 0 (0 - 0) | 0 | 0 (0 - 0) | 0 | 0 (0 - 0) | 0 | 0 (0 - 0) | 0 | 0 (0 - 0) |
| Septicemia | 46 | 11.16 (8.17 - 14.89) ^b^ | 51 | 5.24 (3.9 - 6.88) ^b^ | 33 | 4.47 (3.08 - 6.28) ^b^ | 24 | 6.98 (4.48 - 10.39) ^b^ | 154 | 6.24 (5.29 - 7.31) ^b^ |
| Other Infectious and Parasitic Diseases including HIV | 30 | 14.13 (9.53 - 20.17) ^b^ | 36 | 7.58 (5.31 - 10.49) ^b^ | 24 | 7.84 (5.02 - 11.67) ^b^ | 10 | 7.07 (3.39 - 13.01) ^b^ | 100 | 8.81 (7.17 - 10.72) ^b^ |
| Diabetes Mellitus | 25 | 5.17 (3.34 - 7.63) ^b^ | 69 | 4.39 (3.42 - 5.56) ^b^ | 47 | 3.15 (2.32 - 4.19) ^b^ | 38 | 6.42 (4.55 - 8.82) ^b^ | 179 | 4.33 (3.71 - 5.01) ^b^ |
| Alzheimer’s (ICD-9 and 10 only) | 8 | 0.96 (0.41 - 1.88) | 42 | 1.34 (0.97 - 1.81) | 51 | 2.32 (1.73 - 3.05) ^b^ | 27 | 3.32 (2.19 - 4.83) ^b^ | 128 | 1.83 (1.53 - 2.18) ^b^ |
| Diseases of Heart | 514 | 5.95 (5.45 - 6.49) ^b^ | 904 | 3.51 (3.29 - 3.75) ^b^ | 674 | 3.87 (3.58 - 4.17) ^b^ | 363 | 5.69 (5.12 - 6.31) ^b^ | 2,455 | 4.22 (4.06 - 4.39) ^b^ |
| Hypertension without Heart Disease | 15 | 4.45 (2.49 - 7.33) ^b^ | 26 | 2.59 (1.69 - 3.8) ^b^ | 24 | 2.83 (1.81 - 4.21) ^b^ | 13 | 5.43 (2.89 - 9.28) ^b^ | 78 | 3.21 (2.54 - 4.01) ^b^ |
| Cerebrovascular Diseases | 54 | 3.33 (2.5 - 4.35) ^b^ | 175 | 3.48 (2.99 - 4.04) ^b^ | 152 | 4.31 (3.66 - 5.06) ^b^ | 73 | 6.27 (4.91 - 7.88) ^b^ | 454 | 4.01 (3.65 - 4.39) ^b^ |
| Atherosclerosis | 5 | 4.84 (1.57 - 11.29) ^b^ | 11 | 3.81 (1.9 - 6.82) ^b^ | 10 | 8.31 (3.98 - 15.27) ^b^ | 0 | 0 (0 - 0) | 26 | 5.08 (3.32 - 7.44) ^b^ |
| Aortic Aneurysm and Dissection | 8 | 4.56 (1.97 - 8.98) ^b^ | 26 | 4.58 (2.99 - 6.72) ^b^ | 19 | 5.17 (3.11 - 8.07) ^b^ | 10 | 8.78 (4.21 - 16.15) ^b^ | 63 | 5.15 (3.95 - 6.58) ^b^ |
| Other Diseases of Arteries, Arterioles, Capillaries | 14 | 11.91 (6.51 - 19.99) ^b^ | 20 | 6.87 (4.2 - 10.61) ^b^ | 11 | 5.52 (2.75 - 9.87) ^b^ | 4 | 18.63 (5.08 - 47.71) ^b^ | 49 | 7.78 (5.76 - 10.29) ^b^ |
| Pneumonia and Influenza | 54 | 5.01 (3.76 - 6.54) ^b^ | 92 | 2.91 (2.35 - 3.57) ^b^ | 69 | 3.83 (2.98 - 4.85) ^b^ | 41 | 6.77 (4.85 - 9.18) ^b^ | 256 | 3.85 (3.4 - 4.36) ^b^ |
| Chronic Obstructive Pulmonary Disease and Allied Cond | 212 | 5.69 (4.95 - 6.51) ^b^ | 462 | 3.93 (3.58 - 4.3) ^b^ | 389 | 4.96 (4.48 - 5.48) ^b^ | 205 | 7.09 (6.16 - 8.14) ^b^ | 1,268 | 4.84 (4.57 - 5.11) ^b^ |
| Stomach and Duodenal Ulcers | 6 | 8.16 (2.99 - 17.76) ^b^ | 8 | 3.39 (1.47 - 6.69) ^b^ | 6 | 3.95 (1.45 - 8.59) ^b^ | 2 | 4.65 (0.56 - 16.78) | 22 | 4.36 (2.73 - 6.6) ^b^ |
| Chronic Liver Disease and Cirrhosis | 26 | 16.12 (10.53 - 23.62) ^b^ | 54 | 11.47 (8.62 - 14.97) ^b^ | 29 | 8.94 (5.98 - 12.83) ^b^ | 11 | 16.3 (8.14 - 29.17) ^b^ | 120 | 11.72 (9.72 - 14.01) ^b^ |
| Nephritis, Nephrotic Syndrome and Nephrosis | 17 | 2.68 (1.56 - 4.29) ^b^ | 55 | 2.63 (1.98 - 3.43) ^b^ | 39 | 3.04 (2.16 - 4.16) ^b^ | 19 | 4.7 (2.83 - 7.34) ^b^ | 130 | 2.95 (2.46 - 3.5) ^b^ |
| Complications of Pregnancy, Childbirth, Puerperium | 0 | 0 (0 - 4067.39) | 1 | 457.03 (11.57 - 2546.39) ^b^ | 0 | 0 (0 - 0) | 0 | 0 (0 - 0) | 1 | 323.1 (8.18 - 1800.21) ^b^ |
| Congenital Anomalies | 1 | 14.87 (0.38 - 82.88) | 0 | 0 (0 - 10.47) | 2 | 3.9 (0.47 - 14.11) | 1 | 133.48 (3.38 - 743.72) ^b^ | 4 | 4.26 (1.16 - 10.9) ^b^ |
| Certain Conditions Originating in Perinatal Period | 0 | 0 (0 - 0) | 0 | 0 (0 - 0) | 0 | 0 (0 - 0) | 0 | 0 (0 - 0) | 0 | 0 (0 - 0) |
| Symptoms, Signs, and Ill-Defined Conditions | 21 | 6.28 (3.89 - 9.6) ^b^ | 41 | 3.4 (2.44 - 4.61) ^b^ | 35 | 3 (2.09 - 4.17) ^b^ | 14 | 4.06 (2.22 - 6.8) ^b^ | 111 | 3.64 (2.99 - 4.38) ^b^ |
| Accidents and Adverse Effects | 62 | 7.62 (5.84 - 9.76) ^b^ | 125 | 5.14 (4.28 - 6.13) ^b^ | 84 | 6.1 (4.87 - 7.55) ^b^ | 44 | 10.75 (7.81 - 14.43) ^b^ | 315 | 6.26 (5.59 - 6.99) ^b^ |
| Suicide and Self-Inflicted Injury | 39 | 14.11 (10.03 - 19.29) ^b^ | 61 | 9.89 (7.56 - 12.7) ^b^ | 27 | 10.06 (6.63 - 14.64) ^b^ | 7 | 7.76 (3.12 - 15.98) ^b^ | 134 | 10.7 (8.97 - 12.68) ^b^ |
| Homicide and Legal Intervention | 3 | 17.94 (3.7 - 52.42) ^b^ | 6 | 16.09 (5.9 - 35.01) ^b^ | 3 | 13.41 (2.76 - 39.18) ^b^ | 1 | 2.22 (0.06 - 12.39) | 13 | 10.71 (5.7 - 18.32) ^b^ |
| Other Cause of Death | 220 | 5.04 (4.4 - 5.75) ^b^ | 433 | 3.11 (2.82 - 3.42) ^b^ | 380 | 3.75 (3.39 - 4.15) ^b^ | 215 | 5.32 (4.63 - 6.08) ^b^ | 1,248 | 3.85 (3.63 - 4.06) ^b^ |

**1** number of cancer patients who died due to each cause of death **2** 95% Confidence interval **b** P-value < 0.05

| **Supplementary Table 7.** Standardized-mortality ratios (SMRs) for each cause of death following laryngeal cancer diagnosis in Black patients. | | | | | | | | | | |
| --- | --- | --- | --- | --- | --- | --- | --- | --- | --- | --- |
| **Cause of death** | <1 year | | 1-5 years | | 5-10 years | | >10 years | | Total | |
|  | Observed^1^ | SMR (95% CI^2^) | Observed^1^ | SMR (95% CI^2^) | Observed^1^ | SMR (95% CI^2^) | Observed^1^ | SMR (95% CI^2^) | Observed^1^ | SMR (95% CI^2^) |
| All causes of death | 1,391 | 14.33 (13.59 - 15.1) ^b^ | 1,922 | 9.13 (8.73 - 9.55) ^b^ | 681 | 5.94 (5.51 - 6.41) ^b^ | 312 | 8.1 (7.22 - 9.05) ^b^ | 4,306 | 9.35 (9.07 - 9.63) ^b^ |
| Laryngeal cancer | 618 | 19.41 (17.91 - 21.01) ^b^ | 805 | 18.06 (16.84 - 19.35) ^b^ | 111 | 7.86 (6.47 - 9.47) ^b^ | 47 | 10.48 (7.7 - 13.94) ^b^ | 1,581 | 16.64 (15.83 - 17.48) ^b^ |
| Other non-laryngeal cancer causes of death | 410 | 13.97 (12.65 - 15.39) ^b^ | 647 | 9.88 (9.13 - 10.67) ^b^ | 234 | 6.57 (5.76 - 7.47) ^b^ | 108 | 8.49 (6.97 - 10.25) ^b^ | 1,399 | 9.77 (9.26 - 10.3) ^b^ |
| All non-cancer causes of death | 363 | 10.12 (9.1 - 11.22) ^b^ | 470 | 4.68 (4.27 - 5.13) ^b^ | 336 | 5.18 (4.64 - 5.77) ^b^ | 157 | 7.36 (6.26 - 8.61) ^b^ | 1,326 | 5.96 (5.65 - 6.29) ^b^ |
| In situ, benign or unknown behavior neoplasm | 7 | 24.99 (10.05 - 51.49) ^b^ | 11 | 17.48 (8.73 - 31.28) ^b^ | 3 | 26.41 (5.45 - 77.18) ^b^ | 0 | 0 (0 - 0) | 21 | 20.53 (12.71 - 31.38) ^b^ |
| Tuberculosis | 0 | 0 (0 - 177.52) | 1 | 13.79 (0.35 - 76.83) | 0 | 0 (0 - 0) | 0 | 0 (0 - 0) | 1 | 10.72 (0.27 - 59.72) |
| Syphilis | 0 | 0 (0 - 0) | 0 | 0 (0 - 0) | 0 | 0 (0 - 0) | 0 | 0 (0 - 0) | 0 | 0 (0 - 0) |
| Septicemia | 22 | 17.58 (11.02 - 26.61) ^b^ | 26 | 10.07 (6.58 - 14.76) ^b^ | 10 | 7.07 (3.39 - 13) ^b^ | 5 | 36.66 (11.9 - 85.55) ^b^ | 63 | 11.7 (8.99 - 14.97) ^b^ |
| Other Infectious and Parasitic Diseases including HIV | 26 | 45.79 (29.91 - 67.1) ^b^ | 17 | 12.83 (7.47 - 20.54) ^b^ | 12 | 21.93 (11.33 - 38.3) ^b^ | 3 | 58.16 (11.99 - 169.97) ^b^ | 58 | 23.28 (17.68 - 30.09) ^b^ |
| Diabetes Mellitus | 12 | 10.54 (5.45 - 18.42) ^b^ | 8 | 2.12 (0.91 - 4.17) | 16 | 6.97 (3.99 - 11.32) ^b^ | 6 | 7.54 (2.77 - 16.41) ^b^ | 42 | 5.24 (3.78 - 7.09) ^b^ |
| Alzheimer’s (ICD-9 and 10 only) | 1 | 0.95 (0.02 - 5.29) | 4 | 1.1 (0.3 - 2.82) | 7 | 2.2 (0.89 - 4.54) | 4 | 24.65 (6.72 - 63.11) ^b^ | 16 | 1.99 (1.14 - 3.24) ^b^ |
| Diseases of Heart | 120 | 9.97 (8.27 - 11.93) ^b^ | 166 | 5.1 (4.35 - 5.94) ^b^ | 102 | 5.15 (4.2 - 6.25) ^b^ | 48 | 6.81 (5.02 - 9.04) ^b^ | 436 | 6.1 (5.54 - 6.7) ^b^ |
| Hypertension without Heart Disease | 7 | 8.92 (3.59 - 18.38) ^b^ | 12 | 7.37 (3.81 - 12.87) ^b^ | 5 | 4.81 (1.56 - 11.24) ^b^ | 1 | 46.26 (1.17 - 257.73) ^b^ | 25 | 7.2 (4.66 - 10.63) ^b^ |
| Cerebrovascular Diseases | 11 | 5.57 (2.78 - 9.97) ^b^ | 30 | 5.36 (3.61 - 7.65) ^b^ | 17 | 5.27 (3.07 - 8.43) ^b^ | 14 | 13.94 (7.62 - 23.38) ^b^ | 72 | 6.1 (4.77 - 7.68) ^b^ |
| Atherosclerosis | 2 | 110.93 (13.43 - 400.7) ^b^ | 0 | 0 (0 - 0) | 0 | 0 (0 - 0) | 0 | 0 (0 - 0) | 2 | 110.93 (13.43 - 400.7) ^b^ |
| Aortic Aneurysm and Dissection | 1 | 6.8 (0.17 - 37.87) | 2 | 4.69 (0.57 - 16.96) | 3 | 5.4 (1.11 - 15.77) ^b^ | 0 | 0 (0 - 0) | 6 | 5.31 (1.95 - 11.57) ^b^ |
| Other Diseases of Arteries, Arterioles, Capillaries | 3 | 10.65 (2.2 - 31.11) ^b^ | 4 | 5.95 (1.62 - 15.24) ^b^ | 4 | 20.51 (5.59 - 52.51) ^b^ | 0 | 0 (0 - 0) | 11 | 9.57 (4.78 - 17.13) ^b^ |
| Pneumonia and Influenza | 10 | 7.21 (3.46 - 13.26) ^b^ | 23 | 7.55 (4.79 - 11.33) ^b^ | 10 | 4.33 (2.08 - 7.96) ^b^ | 8 | 6.09 (2.63 - 12) ^b^ | 51 | 6.33 (4.71 - 8.32) ^b^ |
| Chronic Obstructive Pulmonary Disease and Allied Cond | 40 | 10.46 (7.47 - 14.24) ^b^ | 41 | 4.12 (2.96 - 5.59) ^b^ | 32 | 4.82 (3.3 - 6.81) ^b^ | 17 | 7.2 (4.19 - 11.52) ^b^ | 130 | 5.71 (4.77 - 6.78) ^b^ |
| Stomach and Duodenal Ulcers | 0 | 0 (0 - 60.1) | 3 | 31 (6.39 - 90.59) ^b^ | 0 | 0 (0 - 0) | 0 | 0 (0 - 0) | 3 | 18.97 (3.91 - 55.44) ^b^ |
| Chronic Liver Disease and Cirrhosis | 6 | 16.25 (5.96 - 35.36) ^b^ | 8 | 10.69 (4.61 - 21.06) ^b^ | 6 | 22.52 (8.26 - 49.01) ^b^ | 1 | 33.9 (0.86 - 188.85) | 21 | 14.85 (9.19 - 22.7) ^b^ |
| Nephritis, Nephrotic Syndrome and Nephrosis | 9 | 5.62 (2.57 - 10.67) ^b^ | 10 | 1.75 (0.84 - 3.22) | 18 | 4.87 (2.88 - 7.69) ^b^ | 5 | 8 (2.6 - 18.66) ^b^ | 42 | 3.61 (2.6 - 4.88) ^b^ |
| Complications of Pregnancy, Childbirth, Puerperium | 0 | 0 (0 - 0) | 0 | 0 (0 - 0) | 0 | 0 (0 - 0) | 0 | 0 (0 - 0) | 0 | 0 (0 - 0) |
| Congenital Anomalies | 0 | 0 (0 - 97.71) | 2 | 28.75 (3.48 - 103.86) ^b^ | 0 | 0 (0 - 0) | 0 | 0 (0 - 0) | 2 | 18.64 (2.26 - 67.32) ^b^ |
| Certain Conditions Originating in Perinatal Period | 0 | 0 (0 - 0) | 0 | 0 (0 - 0) | 0 | 0 (0 - 0) | 0 | 0 (0 - 0) | 0 | 0 (0 - 0) |
| Symptoms, Signs, and Ill-Defined Conditions | 13 | 11.82 (6.29 - 20.21) ^b^ | 9 | 2.37 (1.08 - 4.5) ^b^ | 15 | 4.62 (2.58 - 7.61) ^b^ | 4 | 8.54 (2.33 - 21.86) ^b^ | 41 | 4.76 (3.42 - 6.46) ^b^ |
| Accidents and Adverse Effects | 11 | 11.49 (5.74 - 20.57) ^b^ | 22 | 7.75 (4.86 - 11.74) ^b^ | 13 | 9.39 (5 - 16.06) ^b^ | 3 | 16.74 (3.45 - 48.92) ^b^ | 49 | 9.14 (6.77 - 12.09) ^b^ |
| Suicide and Self-Inflicted Injury | 2 | 13.74 (1.66 - 49.64) ^b^ | 2 | 4.27 (0.52 - 15.41) | 3 | 47.59 (9.81 - 139.08) ^b^ | 0 | 0 (0 - 0) | 7 | 10.33 (4.15 - 21.29) ^b^ |
| Homicide and Legal Intervention | 1 | 5.57 (0.14 - 31.01) | 5 | 17.48 (5.68 - 40.8) ^b^ | 0 | 0 (0 - 0) | 0 | 0 (0 - 0) | 6 | 12.88 (4.73 - 28.04) ^b^ |
| Other Cause of Death | 59 | 8.86 (6.74 - 11.43) ^b^ | 64 | 3.13 (2.41 - 4) ^b^ | 60 | 4.04 (3.08 - 5.2) ^b^ | 38 | 5.33 (3.77 - 7.32) ^b^ | 221 | 4.5 (3.93 - 5.14) ^b^ |

**1** number of cancer patients who died due to each cause of death **2** 95% Confidence interval **b** P-value < 0.05

| **Supplementary Table 8.** Standardized-mortality ratios (SMRs) for each cause of death following laryngeal cancer diagnosis in Asian or Pacific Islander patients. | | | | | | | | | | |
| --- | --- | --- | --- | --- | --- | --- | --- | --- | --- | --- |
| **Cause of death** | <1 year | | 1-5 years | | 5-10 years | | >10 years | | Total | |
|  | Observed^1^ | SMR (95% CI^2^) | Observed^1^ | SMR (95% CI^2^) | Observed^1^ | SMR (95% CI^2^) | Observed^1^ | SMR (95% CI^2^) | Observed^1^ | SMR (95% CI^2^) |
| All causes of death | 186 | 12.97 (11.17 - 14.97) ^b^ | 292 | 8.58 (7.62 - 9.62) ^b^ | 117 | 5.45 (4.51 - 6.53) ^b^ | 61 | 7.45 (5.7 - 9.58) ^b^ | 656 | 8.41 (7.78 - 9.08) ^b^ |
| Laryngeal cancer | 108 | 21.71 (17.81 - 26.21) ^b^ | 144 | 23.4 (19.74 - 27.55) ^b^ | 22 | 20.51 (12.85 - 31.05) ^b^ | 4 | 63.58 (17.32 - 162.79) ^b^ | 278 | 22.67 (20.08 - 25.5) ^b^ |
| Other non-laryngeal cancer causes of death | 45 | 12.03 (8.78 - 16.1) ^b^ | 88 | 9.36 (7.51 - 11.53) ^b^ | 46 | 7.33 (5.37 - 9.77) ^b^ | 21 | 12.68 (7.85 - 19.39) ^b^ | 200 | 9.49 (8.22 - 10.9) ^b^ |
| All non-cancer causes of death | 33 | 5.86 (4.04 - 8.23) ^b^ | 60 | 3.25 (2.48 - 4.18) ^b^ | 49 | 3.47 (2.57 - 4.59) ^b^ | 36 | 5.57 (3.9 - 7.71) ^b^ | 178 | 3.98 (3.42 - 4.61) ^b^ |
| In situ, benign or unknown behavior neoplasm | 0 | 0 (0 - 16.48) | 2 | 3.85 (0.47 - 13.91) | 1 | 4.76 (0.12 - 26.51) | 0 | 0 (0 - 0) | 3 | 3.15 (0.65 - 9.19) |
| Tuberculosis | 0 | 0 (0 - 0) | 0 | 0 (0 - 0) | 0 | 0 (0 - 0) | 0 | 0 (0 - 0) | 0 | 0 (0 - 0) |
| Syphilis | 0 | 0 (0 - 0) | 0 | 0 (0 - 0) | 0 | 0 (0 - 0) | 0 | 0 (0 - 0) | 0 | 0 (0 - 0) |
| Septicemia | 1 | 26.2 (0.66 - 145.96) | 0 | 0 (0 - 25.43) | 2 | 17.76 (2.15 - 64.16) ^b^ | 0 | 0 (0 - 0) | 3 | 10.14 (2.09 - 29.64) ^b^ |
| Other Infectious and Parasitic Diseases including HIV | 0 | 0 (0 - 33.74) | 0 | 0 (0 - 7.84) | 1 | 1.88 (0.05 - 10.46) | 1 | 9.29 (0.24 - 51.77) | 2 | 1.64 (0.2 - 5.92) |
| Diabetes Mellitus | 1 | 4.05 (0.1 - 22.55) | 1 | 0.83 (0.02 - 4.61) | 4 | 6.4 (1.74 - 16.38) ^b^ | 0 | 0 (0 - 0) | 6 | 2.88 (1.06 - 6.27) ^b^ |
| Alzheimer’s (ICD-9 and 10 only) | 0 | 0 (0 - 282.83) | 0 | 0 (0 - 67.03) | 0 | 0 (0 - 38.91) | 1 | 4.27 (0.11 - 23.77) | 1 | 2.52 (0.06 - 14.03) |
| Diseases of Heart | 9 | 4.54 (2.08 - 8.62) ^b^ | 21 | 3.78 (2.34 - 5.78) ^b^ | 14 | 3 (1.64 - 5.03) ^b^ | 14 | 5.9 (3.22 - 9.89) ^b^ | 58 | 3.98 (3.02 - 5.15) ^b^ |
| Hypertension without Heart Disease | 1 | 13.29 (0.34 - 74.07) | 1 | 3.01 (0.08 - 16.78) | 2 | 6.27 (0.76 - 22.67) | 1 | 4.67 (0.12 - 26.03) | 5 | 5.32 (1.73 - 12.41) ^b^ |
| Cerebrovascular Diseases | 2 | 3.9 (0.47 - 14.07) | 11 | 7.68 (3.83 - 13.74) ^b^ | 4 | 10.52 (2.87 - 26.95) ^b^ | 1 | 8.76 (0.22 - 48.8) | 18 | 7.38 (4.37 - 11.66) ^b^ |
| Atherosclerosis | 0 | 0 (0 - 0) | 0 | 0 (0 - 0) | 0 | 0 (0 - 0) | 0 | 0 (0 - 0) | 0 | 0 (0 - 0) |
| Aortic Aneurysm and Dissection | 0 | 0 (0 - 189.88) | 1 | 20.2 (0.51 - 112.56) | 0 | 0 (0 - 0) | 0 | 0 (0 - 0) | 1 | 14.51 (0.37 - 80.83) |
| Other Diseases of Arteries, Arterioles, Capillaries | 0 | 0 (0 - 0) | 0 | 0 (0 - 0) | 0 | 0 (0 - 0) | 0 | 0 (0 - 0) | 0 | 0 (0 - 0) |
| Pneumonia and Influenza | 2 | 5.04 (0.61 - 18.19) | 4 | 2.79 (0.76 - 7.13) | 4 | 3.77 (1.03 - 9.66) ^b^ | 3 | 4.87 (1 - 14.24) ^b^ | 13 | 3.7 (1.97 - 6.34) ^b^ |
| Chronic Obstructive Pulmonary Disease and Allied Cond | 1 | 1.89 (0.05 - 10.55) | 6 | 3.51 (1.29 - 7.64) ^b^ | 4 | 3.52 (0.96 - 9.01) | 3 | 10.72 (2.21 - 31.33) ^b^ | 14 | 3.83 (2.09 - 6.43) ^b^ |
| Stomach and Duodenal Ulcers | 1 | 25.8 (0.65 - 143.76) | 1 | 5.7 (0.14 - 31.77) | 0 | 0 (0 - 0) | 0 | 0 (0 - 0) | 2 | 9.34 (1.13 - 33.74) ^b^ |
| Chronic Liver Disease and Cirrhosis | 1 | 77.02 (1.95 - 429.13) ^b^ | 1 | 44.82 (1.13 - 249.71) ^b^ | 0 | 0 (0 - 0) | 0 | 0 (0 - 0) | 2 | 56.66 (6.86 - 204.69) ^b^ |
| Nephritis, Nephrotic Syndrome and Nephrosis | 0 | 0 (0 - 0) | 0 | 0 (0 - 0) | 0 | 0 (0 - 0) | 0 | 0 (0 - 0) | 0 | 0 (0 - 0) |
| Complications of Pregnancy, Childbirth, Puerperium | 0 | 0 (0 - 0) | 0 | 0 (0 - 0) | 0 | 0 (0 - 0) | 0 | 0 (0 - 0) | 0 | 0 (0 - 0) |
| Congenital Anomalies | 0 | 0 (0 - 37.19) | 1 | 3.57 (0.09 - 19.87) | 0 | 0 (0 - 0) | 0 | 0 (0 - 0) | 1 | 2.63 (0.07 - 14.68) |
| Certain Conditions Originating in Perinatal Period | 0 | 0 (0 - 0) | 0 | 0 (0 - 0) | 0 | 0 (0 - 0) | 0 | 0 (0 - 0) | 0 | 0 (0 - 0) |
| Symptoms, Signs, and Ill-Defined Conditions | 1 | 21.3 (0.54 - 118.68) | 1 | 5.72 (0.14 - 31.85) | 0 | 0 (0 - 12.69) | 2 | 15.99 (1.94 - 57.76) ^b^ | 4 | 6.27 (1.71 - 16.06) ^b^ |
| Accidents and Adverse Effects | 2 | 6.04 (0.73 - 21.82) | 1 | 0.78 (0.02 - 4.35) | 2 | 1.77 (0.21 - 6.4) | 1 | 20.33 (0.51 - 113.26) | 6 | 2.15 (0.79 - 4.68) |
| Suicide and Self-Inflicted Injury | 1 | 310.42 (7.86 - 1729.55) ^b^ | 0 | 0 (0 - 0) | 0 | 0 (0 - 0) | 0 | 0 (0 - 0) | 1 | 310.42 (7.86 - 1729.55) ^b^ |
| Homicide and Legal Intervention | 0 | 0 (0 - 0) | 0 | 0 (0 - 0) | 0 | 0 (0 - 0) | 0 | 0 (0 - 0) | 0 | 0 (0 - 0) |
| Other Cause of Death | 10 | 10.54 (5.05 - 19.39) ^b^ | 8 | 2.2 (0.95 - 4.33) | 11 | 3.09 (1.54 - 5.52) ^b^ | 9 | 3.83 (1.75 - 7.27) ^b^ | 38 | 3.62 (2.56 - 4.97) ^b^ |

**1** number of cancer patients who died due to each cause of death **2** 95% Confidence interval **b** P-value < 0.05

| **Supplementary Table 9.** Standardized-mortality ratios (SMRs) for each cause of death following laryngeal cancer diagnosis in Indian American/Alaska Native patients. | | | | | | | | | | |
| --- | --- | --- | --- | --- | --- | --- | --- | --- | --- | --- |
| **Cause of death** | <1 year | | 1-5 years | | 5-10 years | | >10 years | | Total | |
|  | Observed^1^ | SMR (95% CI^2^) | Observed^1^ | SMR (95% CI^2^) | Observed^1^ | SMR (95% CI^2^) | Observed^1^ | SMR (95% CI^2^) | Observed^1^ | SMR (95% CI^2^) |
| All causes of death | 29 | 25.43 (17.03 - 36.53) ^b^ | 47 | 15.27 (11.22 - 20.3) ^b^ | 26 | 11.65 (7.61 - 17.07) ^b^ | 7 | 15.95 (6.41 - 32.86) ^b^ | 109 | 15.82 (12.99 - 19.09) ^b^ |
| Laryngeal cancer | 14 | 37.11 (20.29 - 62.26) ^b^ | 22 | 55.21 (34.6 - 83.59) ^b^ | 4 | 100.22 (27.31 - 256.61) ^b^ | 0 | 0 (0 - 0) | 40 | 49.04 (35.03 - 66.78) ^b^ |
| Other non-laryngeal cancer causes of death | 10 | 28.44 (13.64 - 52.29) ^b^ | 16 | 16.82 (9.61 - 27.31) ^b^ | 7 | 12.35 (4.96 - 25.44) ^b^ | 5 | 12.55 (4.07 - 29.28) ^b^ | 38 | 16.75 (11.85 - 22.99) ^b^ |
| All non-cancer causes of death | 5 | 12.16 (3.95 - 28.37) ^b^ | 9 | 5.21 (2.38 - 9.88) ^b^ | 15 | 9.23 (5.17 - 15.23) ^b^ | 2 | 49.36 (5.98 - 178.3) ^b^ | 31 | 8.15 (5.54 - 11.56) ^b^ |
| In situ, benign or unknown behavior neoplasm | 0 | 0 (0 - 0) | 0 | 0 (0 - 0) | 0 | 0 (0 - 0) | 0 | 0 (0 - 0) | 0 | 0 (0 - 0) |
| Tuberculosis | 0 | 0 (0 - 0) | 0 | 0 (0 - 0) | 0 | 0 (0 - 0) | 0 | 0 (0 - 0) | 0 | 0 (0 - 0) |
| Syphilis | 0 | 0 (0 - 0) | 0 | 0 (0 - 0) | 0 | 0 (0 - 0) | 0 | 0 (0 - 0) | 0 | 0 (0 - 0) |
| Septicemia | 1 | 519.99 (13.17 - 2897.21) ^b^ | 0 | 0 (0 - 0) | 0 | 0 (0 - 0) | 0 | 0 (0 - 0) | 1 | 519.99 (13.17 - 2897.21) ^b^ |
| Other Infectious and Parasitic Diseases including HIV | 0 | 0 (0 - 1025.77) | 0 | 0 (0 - 217.18) | 1 | 183.6 (4.65 - 1022.96) ^b^ | 0 | 0 (0 - 0) | 1 | 38.42 (0.97 - 214.06) |
| Diabetes Mellitus | 0 | 0 (0 - 0) | 0 | 0 (0 - 0) | 0 | 0 (0 - 0) | 0 | 0 (0 - 0) | 0 | 0 (0 - 0) |
| Alzheimer’s (ICD-9 and 10 only) | 0 | 0 (0 - 0) | 0 | 0 (0 - 0) | 0 | 0 (0 - 0) | 0 | 0 (0 - 0) | 0 | 0 (0 - 0) |
| Diseases of Heart | 1 | 26.34 (0.67 - 146.76) | 1 | 5.92 (0.15 - 32.99) | 2 | 18.13 (2.2 - 65.48) ^b^ | 0 | 0 (0 - 0) | 4 | 12.61 (3.44 - 32.29) ^b^ |
| Hypertension without Heart Disease | 1 | 188.9 (4.78 - 1052.5) ^b^ | 0 | 0 (0 - 0) | 0 | 0 (0 - 0) | 0 | 0 (0 - 0) | 1 | 188.9 (4.78 - 1052.5) ^b^ |
| Cerebrovascular Diseases | 1 | 34.52 (0.87 - 192.31) | 1 | 8.39 (0.21 - 46.76) | 1 | 25.14 (0.64 - 140.06) | 0 | 0 (0 - 0) | 3 | 15.97 (3.29 - 46.66) ^b^ |
| Atherosclerosis | 0 | 0 (0 - 0) | 0 | 0 (0 - 0) | 0 | 0 (0 - 0) | 0 | 0 (0 - 0) | 0 | 0 (0 - 0) |
| Aortic Aneurysm and Dissection | 0 | 0 (0 - 0) | 0 | 0 (0 - 0) | 0 | 0 (0 - 0) | 0 | 0 (0 - 0) | 0 | 0 (0 - 0) |
| Other Diseases of Arteries, Arterioles, Capillaries | 0 | 0 (0 - 0) | 0 | 0 (0 - 0) | 0 | 0 (0 - 0) | 0 | 0 (0 - 0) | 0 | 0 (0 - 0) |
| Pneumonia and Influenza | 0 | 0 (0 - 200.02) | 0 | 0 (0 - 38.51) | 0 | 0 (0 - 22.87) | 2 | 49.36 (5.98 - 178.3) ^b^ | 2 | 6.33 (0.77 - 22.86) |
| Chronic Obstructive Pulmonary Disease and Allied Cond | 0 | 0 (0 - 24.49) | 4 | 6.58 (1.79 - 16.85) ^b^ | 4 | 6.91 (1.88 - 17.7) ^b^ | 0 | 0 (0 - 0) | 8 | 5.98 (2.58 - 11.79) ^b^ |
| Stomach and Duodenal Ulcers | 0 | 0 (0 - 0) | 0 | 0 (0 - 0) | 0 | 0 (0 - 0) | 0 | 0 (0 - 0) | 0 | 0 (0 - 0) |
| Chronic Liver Disease and Cirrhosis | 0 | 0 (0 - 437.69) | 0 | 0 (0 - 119.04) | 1 | 34.83 (0.88 - 194.05) | 0 | 0 (0 - 0) | 1 | 14.68 (0.37 - 81.78) |
| Nephritis, Nephrotic Syndrome and Nephrosis | 0 | 0 (0 - 124.25) | 0 | 0 (0 - 22.31) | 1 | 6.41 (0.16 - 35.7) | 0 | 0 (0 - 0) | 1 | 2.85 (0.07 - 15.87) |
| Complications of Pregnancy, Childbirth, Puerperium | 0 | 0 (0 - 0) | 0 | 0 (0 - 0) | 0 | 0 (0 - 0) | 0 | 0 (0 - 0) | 0 | 0 (0 - 0) |
| Congenital Anomalies | 0 | 0 (0 - 0) | 0 | 0 (0 - 0) | 0 | 0 (0 - 0) | 0 | 0 (0 - 0) | 0 | 0 (0 - 0) |
| Certain Conditions Originating in Perinatal Period | 0 | 0 (0 - 0) | 0 | 0 (0 - 0) | 0 | 0 (0 - 0) | 0 | 0 (0 - 0) | 0 | 0 (0 - 0) |
| Symptoms, Signs, and Ill-Defined Conditions | 0 | 0 (0 - 437.69) | 0 | 0 (0 - 90.41) | 1 | 21.86 (0.55 - 121.8) | 0 | 0 (0 - 0) | 1 | 10.53 (0.27 - 58.67) |
| Accidents and Adverse Effects | 0 | 0 (0 - 1856.56) | 1 | 0 (0 - 0) | 0 | 0 (0 - 0) | 0 | 0 (0 - 0) | 1 | 503.29 (12.74 - 2804.13) ^b^ |
| Suicide and Self-Inflicted Injury | 0 | 0 (0 - 182) | 0 | 0 (0 - 30.44) | 2 | 15.41 (1.87 - 55.67) ^b^ | 0 | 0 (0 - 0) | 2 | 7.37 (0.89 - 26.63) |
| Homicide and Legal Intervention | 0 | 0 (0 - 0) | 0 | 0 (0 - 0) | 0 | 0 (0 - 0) | 0 | 0 (0 - 0) | 0 | 0 (0 - 0) |
| Other Cause of Death | 1 | 10.46 (0.26 - 58.29) | 2 | 5.53 (0.67 - 19.96) | 2 | 5.43 (0.66 - 19.6) | 0 | 0 (0 - 0) | 5 | 6.05 (1.97 - 14.12) ^b^ |

**1** number of cancer patients who died due to each cause of death **2** 95% Confidence interval **b** P-value < 0.05

| **Supplementary Table 10.** Standardized-mortality ratios (SMRs) for each cause of death following localized laryngeal cancer diagnosis. | | | | | | | | | | |
| --- | --- | --- | --- | --- | --- | --- | --- | --- | --- | --- |
| **Cause of death** | <1 year | | 1-5 years | | 5-10 years | | >10 years | | Total | |
|  | Observed^1^ | SMR (95% CI^2^) | Observed^1^ | SMR (95% CI^2^) | Observed^1^ | SMR (95% CI^2^) | Observed^1^ | SMR (95% CI^2^) | Observed^1^ | SMR (95% CI^2^) |
| All causes of death | 1,959 | 5.01 (4.79 - 5.24) ^b^ | 5,147 | 4.63 (4.5 - 4.75) ^b^ | 3,164 | 4.36 (4.21 - 4.52) ^b^ | 1,595 | 6.09 (5.8 - 6.4) ^b^ | 11,865 | 4.77 (4.68 - 4.85) ^b^ |
| Laryngeal cancer | 821 | 10.44 (9.74 - 11.18) ^b^ | 1,570 | 11.41 (10.85 - 11.99) ^b^ | 396 | 7.25 (6.56 - 8) ^b^ | 125 | 7.52 (6.26 - 8.97) ^b^ | 2,912 | 10.13 (9.77 - 10.51) ^b^ |
| Other non-laryngeal cancer causes of death | 393 | 4.14 (3.74 - 4.57) ^b^ | 1,510 | 5.45 (5.18 - 5.74) ^b^ | 977 | 5.46 (5.13 - 5.82) ^b^ | 486 | 7.87 (7.19 - 8.6) ^b^ | 3,366 | 5.5 (5.31 - 5.68) ^b^ |
| All non-cancer causes of death | 745 | 3.43 (3.19 - 3.69) ^b^ | 2,067 | 2.96 (2.83 - 3.09) ^b^ | 1,791 | 3.64 (3.48 - 3.82) ^b^ | 984 | 5.37 (5.04 - 5.71) ^b^ | 5,587 | 3.51 (3.42 - 3.61) ^b^ |
| In situ, benign or unknown behavior neoplasm | 8 | 3.27 (1.41 - 6.44) ^b^ | 34 | 5.33 (3.69 - 7.44) ^b^ | 16 | 3.4 (1.94 - 5.51) ^b^ | 16 | 5.17 (2.95 - 8.39) ^b^ | 74 | 4.45 (3.49 - 5.58) ^b^ |
| Tuberculosis | 0 | 0 (0 - 0) | 0 | 0 (0 - 0) | 0 | 0 (0 - 0) | 0 | 0 (0 - 0) | 0 | 0 (0 - 0) |
| Syphilis | 0 | 0 (0 - 0) | 0 | 0 (0 - 0) | 0 | 0 (0 - 0) | 0 | 0 (0 - 0) | 0 | 0 (0 - 0) |
| Septicemia | 29 | 7.9 (5.29 - 11.35) ^b^ | 44 | 4.92 (3.58 - 6.61) ^b^ | 21 | 3 (1.86 - 4.59) ^b^ | 19 | 5.83 (3.51 - 9.11) ^b^ | 113 | 4.94 (4.07 - 5.94) ^b^ |
| Other Infectious and Parasitic Diseases including HIV | 15 | 7.32 (4.1 - 12.08) ^b^ | 26 | 5.13 (3.35 - 7.52) ^b^ | 25 | 6.56 (4.24 - 9.68) ^b^ | 12 | 7.88 (4.07 - 13.76) ^b^ | 78 | 6.26 (4.95 - 7.82) ^b^ |
| Diabetes Mellitus | 20 | 4.13 (2.52 - 6.37) ^b^ | 51 | 2.93 (2.18 - 3.86) ^b^ | 52 | 3.37 (2.51 - 4.42) ^b^ | 38 | 6.13 (4.34 - 8.42) ^b^ | 161 | 3.67 (3.12 - 4.28) ^b^ |
| Alzheimer’s (ICD-9 and 10 only) | 2 | 0.25 (0.03 - 0.92) ^b^ | 34 | 1.14 (0.79 - 1.59) | 50 | 2.21 (1.64 - 2.91) ^b^ | 30 | 3.74 (2.52 - 5.33) ^b^ | 116 | 1.7 (1.4 - 2.04) ^b^ |
| Diseases of Heart | 278 | 3.64 (3.22 - 4.09) ^b^ | 717 | 3.02 (2.81 - 3.25) ^b^ | 577 | 3.62 (3.33 - 3.93) ^b^ | 311 | 5.29 (4.72 - 5.91) ^b^ | 1,883 | 3.54 (3.38 - 3.71) ^b^ |
| Hypertension without Heart Disease | 12 | 4.03 (2.08 - 7.04) ^b^ | 21 | 2.25 (1.39 - 3.44) ^b^ | 25 | 3.26 (2.11 - 4.81) ^b^ | 10 | 4.63 (2.22 - 8.51) ^b^ | 68 | 3.07 (2.38 - 3.89) ^b^ |
| Cerebrovascular Diseases | 34 | 2.3 (1.59 - 3.21) ^b^ | 144 | 3.08 (2.6 - 3.63) ^b^ | 125 | 3.79 (3.16 - 4.52) ^b^ | 66 | 5.91 (4.57 - 7.52) ^b^ | 369 | 3.49 (3.15 - 3.87) ^b^ |
| Atherosclerosis | 1 | 1.21 (0.03 - 6.73) | 7 | 2.45 (0.98 - 5.05) | 10 | 8.31 (3.98 - 15.27) ^b^ | 0 | 0 (0 - 0) | 18 | 3.68 (2.18 - 5.82) ^b^ |
| Aortic Aneurysm and Dissection | 5 | 3.71 (1.2 - 8.65) ^b^ | 18 | 4.21 (2.49 - 6.65) ^b^ | 16 | 4.84 (2.77 - 7.86) ^b^ | 9 | 9.23 (4.22 - 17.52) ^b^ | 48 | 4.84 (3.57 - 6.42) ^b^ |
| Other Diseases of Arteries, Arterioles, Capillaries | 3 | 3.32 (0.69 - 9.71) | 15 | 5.95 (3.33 - 9.81) ^b^ | 9 | 4.84 (2.21 - 9.19) ^b^ | 4 | 18.63 (5.08 - 47.71) ^b^ | 31 | 5.64 (3.83 - 8) ^b^ |
| Pneumonia and Influenza | 30 | 3.49 (2.36 - 4.98) ^b^ | 63 | 2.33 (1.79 - 2.98) ^b^ | 50 | 3.03 (2.25 - 3.99) ^b^ | 37 | 5.72 (4.03 - 7.88) ^b^ | 180 | 3.07 (2.64 - 3.55) ^b^ |
| Chronic Obstructive Pulmonary Disease and Allied Cond | 116 | 3.74 (3.09 - 4.49) ^b^ | 338 | 3.36 (3.01 - 3.74) ^b^ | 303 | 4.46 (3.98 - 5) ^b^ | 157 | 6.28 (5.33 - 7.34) ^b^ | 914 | 4.07 (3.81 - 4.35) ^b^ |
| Stomach and Duodenal Ulcers | 4 | 5.8 (1.58 - 14.86) ^b^ | 6 | 2.73 (1 - 5.93) ^b^ | 5 | 3.32 (1.08 - 7.76) ^b^ | 2 | 4.65 (0.56 - 16.78) | 17 | 3.52 (2.05 - 5.64) ^b^ |
| Chronic Liver Disease and Cirrhosis | 5 | 3.79 (1.23 - 8.85) ^b^ | 44 | 10.77 (7.82 - 14.45) ^b^ | 30 | 12.09 (8.16 - 17.26) ^b^ | 5 | 37.63 (12.22 - 87.82) ^b^ | 84 | 10.48 (8.36 - 12.97) ^b^ |
| Nephritis, Nephrotic Syndrome and Nephrosis | 16 | 2.52 (1.44 - 4.09) ^b^ | 42 | 1.98 (1.43 - 2.68) ^b^ | 45 | 3.21 (2.34 - 4.29) ^b^ | 18 | 4.67 (2.77 - 7.39) ^b^ | 121 | 2.66 (2.21 - 3.18) ^b^ |
| Complications of Pregnancy, Childbirth, Puerperium | 0 | 0 (0 - 0) | 0 | 0 (0 - 0) | 0 | 0 (0 - 0) | 0 | 0 (0 - 0) | 0 | 0 (0 - 0) |
| Congenital Anomalies | 0 | 0 (0 - 21.95) | 2 | 3.36 (0.41 - 12.14) | 1 | 2.47 (0.06 - 13.78) | 0 | 0 (0 - 0) | 3 | 2.57 (0.53 - 7.51) |
| Certain Conditions Originating in Perinatal Period | 0 | 0 (0 - 0) | 0 | 0 (0 - 0) | 0 | 0 (0 - 0) | 0 | 0 (0 - 0) | 0 | 0 (0 - 0) |
| Symptoms, Signs, and Ill-Defined Conditions | 11 | 3.35 (1.67 - 6) ^b^ | 26 | 2.05 (1.34 - 3.01) ^b^ | 29 | 2.17 (1.45 - 3.11) ^b^ | 19 | 4.7 (2.83 - 7.35) ^b^ | 85 | 2.55 (2.03 - 3.15) ^b^ |
| Accidents and Adverse Effects | 28 | 4.24 (2.82 - 6.13) ^b^ | 79 | 3.58 (2.83 - 4.46) ^b^ | 67 | 5.55 (4.3 - 7.05) ^b^ | 33 | 12.81 (8.82 - 17.98) ^b^ | 207 | 4.78 (4.15 - 5.48) ^b^ |
| Suicide and Self-Inflicted Injury | 17 | 8.88 (5.18 - 14.23) ^b^ | 40 | 8.95 (6.39 - 12.19) ^b^ | 21 | 11 (6.81 - 16.82) ^b^ | 5 | 7.99 (2.59 - 18.64) ^b^ | 83 | 9.31 (7.41 - 11.54) ^b^ |
| Homicide and Legal Intervention | 3 | 11.06 (2.28 - 32.32) ^b^ | 7 | 14.22 (5.72 - 29.29) ^b^ | 1 | 4.53 (0.11 - 25.27) | 1 | 2.22 (0.06 - 12.39) | 12 | 8.37 (4.32 - 14.62) ^b^ |
| Other Cause of Death | 108 | 2.78 (2.28 - 3.36) ^b^ | 309 | 2.34 (2.08 - 2.61) ^b^ | 313 | 3.09 (2.75 - 3.45) ^b^ | 192 | 4.33 (3.74 - 4.99) ^b^ | 922 | 2.91 (2.73 - 3.1) ^b^ |

**1** number of cancer patients who died due to each cause of death **2** 95% Confidence interval **b** P-value < 0.05

| **Supplementary Table 11.** Standardized-mortality ratios (SMRs) for each cause of death following regional laryngeal cancer diagnosis. | | | | | | | | | | |
| --- | --- | --- | --- | --- | --- | --- | --- | --- | --- | --- |
| **Cause of death** | <1 year | | 1-5 years | | 5-10 years | | >10 years | | Total | |
|  | Observed^1^ | SMR (95% CI^2^) | Observed^1^ | SMR (95% CI^2^) | Observed^1^ | SMR (95% CI^2^) | Observed^1^ | SMR (95% CI^2^) | Observed^1^ | SMR (95% CI^2^) |
| All causes of death | 2,021 | 18.35 (17.56 - 19.17) ^b^ | 3,055 | 15.37 (14.83 - 15.92) ^b^ | 931 | 10.06 (9.42 - 10.72) ^b^ | 324 | 12.94 (11.57 - 14.42) ^b^ | 6,331 | 14.84 (14.48 - 15.21) ^b^ |
| Laryngeal cancer | 944 | 21.96 (20.58 - 23.4) ^b^ | 1,310 | 25.61 (24.25 - 27.04) ^b^ | 165 | 12.05 (10.28 - 14.04) ^b^ | 47 | 26.08 (19.16 - 34.68) ^b^ | 2,466 | 22.49 (21.62 - 23.4) ^b^ |
| Other non-laryngeal cancer causes of death | 606 | 17.15 (15.81 - 18.57) ^b^ | 1,095 | 16.02 (15.08 - 17) ^b^ | 370 | 11.13 (10.02 - 12.32) ^b^ | 108 | 12.45 (10.22 - 15.04) ^b^ | 2,179 | 14.96 (14.34 - 15.61) ^b^ |
| All non-cancer causes of death | 471 | 14.8 (13.5 - 16.2) ^b^ | 650 | 8.19 (7.58 - 8.85) ^b^ | 396 | 8.68 (7.84 - 9.57) ^b^ | 169 | 11.6 (9.92 - 13.49) ^b^ | 1,686 | 9.84 (9.38 - 10.32) ^b^ |
| In situ, benign or unknown behavior neoplasm | 6 | 11.56 (4.24 - 25.17) ^b^ | 14 | 8.76 (4.79 - 14.69) ^b^ | 5 | 9.09 (2.95 - 21.21) ^b^ | 2 | 16.68 (2.02 - 60.25) ^b^ | 27 | 9.68 (6.38 - 14.09) ^b^ |
| Tuberculosis | 1 | 0 (0 - 0) | 0 | 0 (0 - 0) | 0 | 0 (0 - 0) | 0 | 0 (0 - 0) | 1 | 0 (0 - 0) |
| Syphilis | 0 | 0 (0 - 0) | 0 | 0 (0 - 0) | 0 | 0 (0 - 0) | 0 | 0 (0 - 0) | 0 | 0 (0 - 0) |
| Septicemia | 21 | 35.64 (22.06 - 54.48) ^b^ | 15 | 10.07 (5.64 - 16.61) ^b^ | 11 | 14.78 (7.38 - 26.45) ^b^ | 2 | 20.6 (2.49 - 74.4) ^b^ | 49 | 16.78 (12.41 - 22.18) ^b^ |
| Other Infectious and Parasitic Diseases including HIV | 23 | 60.13 (38.12 - 90.22) ^b^ | 14 | 22.32 (12.2 - 37.45) ^b^ | 8 | 33.19 (14.33 - 65.4) ^b^ | 2 | 40.27 (4.88 - 145.46) ^b^ | 47 | 36.14 (26.56 - 48.06) ^b^ |
| Diabetes Mellitus | 6 | 9.28 (3.4 - 20.19) ^b^ | 15 | 6.96 (3.89 - 11.47) ^b^ | 11 | 7.92 (3.96 - 14.18) ^b^ | 2 | 5.37 (0.65 - 19.4) | 34 | 7.45 (5.16 - 10.41) ^b^ |
| Alzheimer’s (ICD-9 and 10 only) | 2 | 4.17 (0.51 - 15.07) | 3 | 1.44 (0.3 - 4.2) | 4 | 2.96 (0.81 - 7.57) | 1 | 2.85 (0.07 - 15.85) | 10 | 2.34 (1.12 - 4.31) ^b^ |
| Diseases of Heart | 160 | 15.62 (13.3 - 18.24) ^b^ | 204 | 8.98 (7.79 - 10.3) ^b^ | 114 | 8.62 (7.11 - 10.35) ^b^ | 46 | 10.07 (7.37 - 13.43) ^b^ | 524 | 10.32 (9.46 - 11.25) ^b^ |
| Hypertension without Heart Disease | 5 | 13.32 (4.32 - 31.08) ^b^ | 12 | 15.09 (7.8 - 26.35) ^b^ | 2 | 5.4 (0.65 - 19.52) | 4 | 17.38 (4.73 - 44.49) ^b^ | 23 | 12.99 (8.23 - 19.49) ^b^ |
| Cerebrovascular Diseases | 16 | 10.4 (5.94 - 16.89) ^b^ | 34 | 7.16 (4.96 - 10) ^b^ | 26 | 9.13 (5.96 - 13.38) ^b^ | 12 | 14.78 (7.64 - 25.82) ^b^ | 88 | 8.84 (7.09 - 10.9) ^b^ |
| Atherosclerosis | 3 | 31.34 (6.46 - 91.6) ^b^ | 3 | 158.55 (32.7 - 463.35) ^b^ | 0 | 0 (0 - 0) | 0 | 0 (0 - 0) | 6 | 52.34 (19.21 - 113.92) ^b^ |
| Aortic Aneurysm and Dissection | 2 | 5.92 (0.72 - 21.39) | 7 | 5.04 (2.03 - 10.38) ^b^ | 3 | 5.12 (1.06 - 14.97) ^b^ | 1 | 6.11 (0.15 - 34.02) | 13 | 5.25 (2.8 - 8.98) ^b^ |
| Other Diseases of Arteries, Arterioles, Capillaries | 8 | 25.47 (11 - 50.19) ^b^ | 3 | 4.36 (0.9 - 12.74) | 6 | 18.14 (6.66 - 39.48) ^b^ | 0 | 0 (0 - 0) | 17 | 12.75 (7.43 - 20.42) ^b^ |
| Pneumonia and Influenza | 15 | 8.07 (4.51 - 13.3) ^b^ | 36 | 7.5 (5.25 - 10.38) ^b^ | 20 | 6.89 (4.21 - 10.65) ^b^ | 9 | 9.1 (4.16 - 17.27) ^b^ | 80 | 7.58 (6.01 - 9.44) ^b^ |
| Chronic Obstructive Pulmonary Disease and Allied Cond | 64 | 12.98 (10 - 16.58) ^b^ | 101 | 8.18 (6.67 - 9.94) ^b^ | 70 | 9.37 (7.3 - 11.83) ^b^ | 34 | 14.15 (9.8 - 19.77) ^b^ | 269 | 9.91 (8.76 - 11.17) ^b^ |
| Stomach and Duodenal Ulcers | 1 | 15.1 (0.38 - 84.14) | 3 | 22.84 (4.71 - 66.74) ^b^ | 0 | 0 (0 - 0) | 0 | 0 (0 - 0) | 4 | 20.24 (5.52 - 51.83) ^b^ |
| Chronic Liver Disease and Cirrhosis | 13 | 31.73 (16.9 - 54.27) ^b^ | 12 | 17.71 (9.15 - 30.93) ^b^ | 2 | 4.52 (0.55 - 16.31) | 4 | 10.2 (2.78 - 26.11) ^b^ | 31 | 16.12 (10.96 - 22.89) ^b^ |
| Nephritis, Nephrotic Syndrome and Nephrosis | 4 | 12.57 (3.43 - 32.2) ^b^ | 8 | 7.56 (3.26 - 14.89) ^b^ | 3 | 5.15 (1.06 - 15.06) ^b^ | 3 | 12.26 (2.53 - 35.84) ^b^ | 18 | 8.17 (4.84 - 12.91) ^b^ |
| Complications of Pregnancy, Childbirth, Puerperium | 0 | 0 (0 - 0) | 0 | 0 (0 - 0) | 0 | 0 (0 - 0) | 0 | 0 (0 - 0) | 0 | 0 (0 - 0) |
| Congenital Anomalies | 0 | 0 (0 - 104.22) | 1 | 9.31 (0.24 - 51.88) | 1 | 9.26 (0.23 - 51.6) | 1 | 133.48 (3.38 - 743.72) ^b^ | 3 | 11.62 (2.4 - 33.95) ^b^ |
| Certain Conditions Originating in Perinatal Period | 0 | 0 (0 - 0) | 0 | 0 (0 - 0) | 0 | 0 (0 - 0) | 0 | 0 (0 - 0) | 0 | 0 (0 - 0) |
| Symptoms, Signs, and Ill-Defined Conditions | 10 | 17.64 (8.46 - 32.44) ^b^ | 13 | 7.82 (4.16 - 13.37) ^b^ | 13 | 25.39 (13.52 - 43.42) ^b^ | 1 | 147.15 (3.73 - 819.87) ^b^ | 37 | 13.46 (9.48 - 18.55) ^b^ |
| Accidents and Adverse Effects | 19 | 14.29 (8.6 - 22.31) ^b^ | 35 | 9.01 (6.27 - 12.53) ^b^ | 20 | 7.5 (4.58 - 11.58) ^b^ | 7 | 5.89 (2.37 - 12.14) ^b^ | 81 | 8.93 (7.09 - 11.1) ^b^ |
| Suicide and Self-Inflicted Injury | 14 | 29.51 (16.13 - 49.5) ^b^ | 15 | 26.03 (14.57 - 42.93) ^b^ | 5 | 29.68 (9.64 - 69.27) ^b^ | 1 | 25.99 (0.66 - 144.8) | 35 | 27.83 (19.38 - 38.7) ^b^ |
| Homicide and Legal Intervention | 1 | 51.14 (1.29 - 284.94) ^b^ | 2 | 30.37 (3.68 - 109.72) ^b^ | 1 | 974.32 (24.67 - 5428.56) ^b^ | 0 | 0 (0 - 0) | 4 | 46.28 (12.61 - 118.5) ^b^ |
| Other Cause of Death | 77 | 12.25 (9.67 - 15.31) ^b^ | 100 | 6.37 (5.18 - 7.75) ^b^ | 71 | 7.76 (6.06 - 9.79) ^b^ | 37 | 14.59 (10.27 - 20.11) ^b^ | 285 | 8.46 (7.51 - 9.51) ^b^ |

**1** number of cancer patients who died due to each cause of death **2** 95% Confidence interval **b** P-value < 0.05

| **Supplementary Table 12.** Standardized-mortality ratios (SMRs) for each cause of death following distant laryngeal cancer diagnosis. | | | | | | | | | | |
| --- | --- | --- | --- | --- | --- | --- | --- | --- | --- | --- |
| **Cause of death** | <1 year | | 1-5 years | | 5-10 years | | >10 years | | Total | |
|  | Observed^1^ | SMR (95% CI^2^) | Observed^1^ | SMR (95% CI^2^) | Observed^1^ | SMR (95% CI^2^) | Observed^1^ | SMR (95% CI^2^) | Observed^1^ | SMR (95% CI^2^) |
| All causes of death | 2,525 | 25.84 (24.85 - 26.87) ^b^ | 2,476 | 16.81 (16.15 - 17.48) ^b^ | 590 | 8.37 (7.71 - 9.07) ^b^ | 237 | 12.21 (10.71 - 13.87) ^b^ | 5,828 | 17.4 (16.96 - 17.85) ^b^ |
| Laryngeal cancer | 1,349 | 30.84 (29.21 - 32.53) ^b^ | 1,219 | 27.86 (26.32 - 29.47) ^b^ | 125 | 11.39 (9.48 - 13.58) ^b^ | 32 | 17.1 (11.69 - 24.13) ^b^ | 2,725 | 27.16 (26.15 - 28.2) ^b^ |
| Other non-laryngeal cancer causes of death | 763 | 26.55 (24.7 - 28.5) ^b^ | 818 | 18.54 (17.29 - 19.86) ^b^ | 209 | 9.58 (8.33 - 10.97) ^b^ | 84 | 12.09 (9.64 - 14.97) ^b^ | 1,874 | 18.44 (17.62 - 19.3) ^b^ |
| All non-cancer causes of death | 413 | 16.38 (14.84 - 18.04) ^b^ | 439 | 7.38 (6.71 - 8.11) ^b^ | 256 | 6.79 (5.98 - 7.68) ^b^ | 121 | 11.43 (9.49 - 13.66) ^b^ | 1,229 | 9.24 (8.73 - 9.78) ^b^ |
| In situ, benign or unknown behavior neoplasm | 7 | 24.49 (9.85 - 50.45) ^b^ | 7 | 10.35 (4.16 - 21.33) ^b^ | 2 | 45.1 (5.46 - 162.93) ^b^ | 0 | 0 (0 - 0) | 16 | 15.9 (9.09 - 25.82) ^b^ |
| Tuberculosis | 0 | 0 (0 - 207.91) | 1 | 30.58 (0.77 - 170.36) | 0 | 0 (0 - 0) | 0 | 0 (0 - 0) | 1 | 19.82 (0.5 - 110.44) |
| Syphilis | 0 | 0 (0 - 0) | 0 | 0 (0 - 0) | 0 | 0 (0 - 0) | 0 | 0 (0 - 0) | 0 | 0 (0 - 0) |
| Septicemia | 17 | 20.46 (11.92 - 32.76) ^b^ | 13 | 9.02 (4.8 - 15.42) ^b^ | 10 | 12.88 (6.18 - 23.68) ^b^ | 6 | 31.85 (11.69 - 69.32) ^b^ | 46 | 14.21 (10.4 - 18.95) ^b^ |
| Other Infectious and Parasitic Diseases including HIV | 14 | 49.17 (26.88 - 82.49) ^b^ | 10 | 16.28 (7.81 - 29.94) ^b^ | 4 | 91.81 (25.01 - 235.06) ^b^ | 0 | 0 (0 - 0) | 28 | 29.7 (19.74 - 42.93) ^b^ |
| Diabetes Mellitus | 8 | 18.2 (7.86 - 35.86) ^b^ | 10 | 21.77 (10.44 - 40.03) ^b^ | 2 | 12.27 (1.49 - 44.31) ^b^ | 1 | 27.63 (0.7 - 153.92) | 21 | 19.12 (11.84 - 29.23) ^b^ |
| Alzheimer’s (ICD-9 and 10 only) | 3 | 5.33 (1.1 - 15.59) ^b^ | 6 | 3.59 (1.32 - 7.82) ^b^ | 1 | 2.54 (0.06 - 14.14) | 0 | 0 (0 - 0) | 10 | 3.81 (1.83 - 7) ^b^ |
| Diseases of Heart | 152 | 16.54 (14.01 - 19.38) ^b^ | 129 | 6.04 (5.04 - 7.18) ^b^ | 76 | 4.74 (3.73 - 5.93) ^b^ | 47 | 9.56 (7.02 - 12.71) ^b^ | 404 | 7.84 (7.1 - 8.65) ^b^ |
| Hypertension without Heart Disease | 3 | 9.02 (1.86 - 26.36) ^b^ | 5 | 9.31 (3.02 - 21.73) ^b^ | 3 | 9.32 (1.92 - 27.23) ^b^ | 0 | 0 (0 - 0) | 11 | 9.23 (4.61 - 16.52) ^b^ |
| Cerebrovascular Diseases | 9 | 7.88 (3.6 - 14.96) ^b^ | 26 | 8.44 (5.52 - 12.37) ^b^ | 18 | 8.24 (4.88 - 13.02) ^b^ | 9 | 13.62 (6.23 - 25.86) ^b^ | 62 | 8.77 (6.73 - 11.25) ^b^ |
| Atherosclerosis | 2 | 36.27 (4.39 - 131.01) ^b^ | 0 | 0 (0 - 0) | 0 | 0 (0 - 0) | 0 | 0 (0 - 0) | 2 | 36.27 (4.39 - 131.01) ^b^ |
| Aortic Aneurysm and Dissection | 0 | 0 (0 - 23.45) | 4 | 15.7 (4.28 - 40.2) ^b^ | 0 | 0 (0 - 0) | 0 | 0 (0 - 0) | 4 | 9.71 (2.64 - 24.86) ^b^ |
| Other Diseases of Arteries, Arterioles, Capillaries | 3 | 22.72 (4.69 - 66.4) ^b^ | 4 | 32.76 (8.92 - 83.87) ^b^ | 0 | 0 (0 - 0) | 0 | 0 (0 - 0) | 7 | 27.54 (11.07 - 56.75) ^b^ |
| Pneumonia and Influenza | 19 | 11.21 (6.75 - 17.5) ^b^ | 18 | 5.7 (3.38 - 9) ^b^ | 12 | 9.15 (4.73 - 15.99) ^b^ | 5 | 22.4 (7.27 - 52.26) ^b^ | 54 | 8.45 (6.35 - 11.03) ^b^ |
| Chronic Obstructive Pulmonary Disease and Allied Cond | 52 | 14.65 (10.94 - 19.21) ^b^ | 56 | 5.4 (4.08 - 7.02) ^b^ | 43 | 6.76 (4.89 - 9.11) ^b^ | 18 | 12.45 (7.38 - 19.67) ^b^ | 169 | 7.78 (6.65 - 9.05) ^b^ |
| Stomach and Duodenal Ulcers | 0 | 0 (0 - 53.94) | 3 | 10.11 (2.09 - 29.55) ^b^ | 1 | 60.25 (1.53 - 335.71) ^b^ | 0 | 0 (0 - 0) | 4 | 10.48 (2.86 - 26.84) ^b^ |
| Chronic Liver Disease and Cirrhosis | 11 | 46.11 (23.02 - 82.5) ^b^ | 7 | 11.51 (4.63 - 23.7) ^b^ | 4 | 10.55 (2.87 - 27.02) ^b^ | 1 | 27.53 (0.7 - 153.39) | 23 | 18.22 (11.55 - 27.34) ^b^ |
| Nephritis, Nephrotic Syndrome and Nephrosis | 4 | 5.52 (1.5 - 14.14) ^b^ | 12 | 4.6 (2.38 - 8.03) ^b^ | 5 | 4.11 (1.33 - 9.59) ^b^ | 3 | 5.26 (1.08 - 15.36) ^b^ | 24 | 4.69 (3 - 6.97) ^b^ |
| Complications of Pregnancy, Childbirth, Puerperium | 0 | 0 (0 - 4067.39) | 1 | 457.03 (11.57 - 2546.39) ^b^ | 0 | 0 (0 - 0) | 0 | 0 (0 - 0) | 1 | 323.1 (8.18 - 1800.21) ^b^ |
| Congenital Anomalies | 1 | 1325.76 (33.57 - 7386.67) ^b^ | 0 | 0 (0 - 0) | 0 | 0 (0 - 0) | 0 | 0 (0 - 0) | 1 | 1325.76 (33.57 - 7386.67) ^b^ |
| Certain Conditions Originating in Perinatal Period | 0 | 0 (0 - 0) | 0 | 0 (0 - 0) | 0 | 0 (0 - 0) | 0 | 0 (0 - 0) | 0 | 0 (0 - 0) |
| Symptoms, Signs, and Ill-Defined Conditions | 8 | 23.66 (10.22 - 46.63) ^b^ | 10 | 12.13 (5.82 - 22.31) ^b^ | 6 | 8.84 (3.24 - 19.24) ^b^ | 0 | 0 (0 - 0) | 24 | 13.03 (8.35 - 19.39) ^b^ |
| Accidents and Adverse Effects | 22 | 19.72 (12.36 - 29.85) ^b^ | 29 | 15.52 (10.4 - 22.3) ^b^ | 11 | 8.29 (4.14 - 14.84) ^b^ | 5 | 13.85 (4.5 - 32.32) ^b^ | 67 | 14.34 (11.11 - 18.21) ^b^ |
| Suicide and Self-Inflicted Injury | 10 | 52.85 (25.35 - 97.2) ^b^ | 6 | 12.24 (4.49 - 26.64) ^b^ | 5 | 11.38 (3.7 - 26.56) ^b^ | 1 | 4.2 (0.11 - 23.41) | 22 | 16.22 (10.16 - 24.55) ^b^ |
| Homicide and Legal Intervention | 0 | 0 (0 - 108.3) | 1 | 25.32 (0.64 - 141.05) | 1 | 451.29 (11.43 - 2514.44) ^b^ | 0 | 0 (0 - 0) | 2 | 26.39 (3.2 - 95.34) ^b^ |
| Other Cause of Death | 68 | 17.78 (13.81 - 22.54) ^b^ | 81 | 9.05 (7.19 - 11.25) ^b^ | 52 | 8.67 (6.48 - 11.37) ^b^ | 25 | 13.12 (8.49 - 19.37) ^b^ | 226 | 10.93 (9.55 - 12.45) ^b^ |

**1** number of cancer patients who died due to each cause of death **2** 95% Confidence interval **b** P-value < 0.05

| **Supplementary Table 13.** Standardized-mortality ratios (SMRs) for each cause of death following laryngeal cancer diagnosis in patients who underwent cancer-directed surgery. | | | | | | | | | | |
| --- | --- | --- | --- | --- | --- | --- | --- | --- | --- | --- |
| **Cause of death** | <1 year | | 1-5 years | | 5-10 years | | >10 years | | Total | |
|  | Observed^1^ | SMR (95% CI^2^) | Observed^1^ | SMR (95% CI^2^) | Observed^1^ | SMR (95% CI^2^) | Observed^1^ | SMR (95% CI^2^) | Observed^1^ | SMR (95% CI^2^) |
| All causes of death | 1,667 | 6.86 (6.53 - 7.19) ^b^ | 4,172 | 6.44 (6.25 - 6.64) ^b^ | 1,951 | 4.75 (4.55 - 4.97) ^b^ | 1,009 | 6.59 (6.19 - 7) ^b^ | 8,799 | 6.05 (5.92 - 6.18) ^b^ |
| Laryngeal cancer | 805 | 13.13 (12.24 - 14.07) ^b^ | 1,617 | 16.63 (15.83 - 17.47) ^b^ | 283 | 7.74 (6.86 - 8.7) ^b^ | 101 | 8.56 (6.97 - 10.4) ^b^ | 2,806 | 13.56 (13.07 - 14.08) ^b^ |
| Other non-laryngeal cancer causes of death | 377 | 6.23 (5.61 - 6.89) ^b^ | 1,266 | 7.84 (7.42 - 8.29) ^b^ | 624 | 6 (5.54 - 6.49) ^b^ | 309 | 8.49 (7.57 - 9.49) ^b^ | 2,576 | 7.11 (6.84 - 7.39) ^b^ |
| All non-cancer causes of death | 485 | 4 (3.65 - 4.37) ^b^ | 1,289 | 3.31 (3.13 - 3.5) ^b^ | 1,044 | 3.87 (3.64 - 4.11) ^b^ | 599 | 5.7 (5.26 - 6.18) ^b^ | 3,417 | 3.86 (3.73 - 3.99) ^b^ |
| In situ, benign or unknown behavior neoplasm | 3 | 2.1 (0.43 - 6.14) | 23 | 6.5 (4.12 - 9.76) ^b^ | 7 | 3.74 (1.5 - 7.71) ^b^ | 8 | 12.36 (5.34 - 24.35) ^b^ | 41 | 5.48 (3.93 - 7.43) ^b^ |
| Tuberculosis | 0 | 0 (0 - 0) | 0 | 0 (0 - 0) | 0 | 0 (0 - 0) | 0 | 0 (0 - 0) | 0 | 0 (0 - 0) |
| Syphilis | 0 | 0 (0 - 0) | 0 | 0 (0 - 0) | 0 | 0 (0 - 0) | 0 | 0 (0 - 0) | 0 | 0 (0 - 0) |
| Septicemia | 21 | 9.83 (6.09 - 15.03) ^b^ | 24 | 3.65 (2.34 - 5.43) ^b^ | 22 | 4.33 (2.71 - 6.56) ^b^ | 12 | 5.49 (2.84 - 9.6) ^b^ | 79 | 4.94 (3.91 - 6.16) ^b^ |
| Other Infectious and Parasitic Diseases including HIV | 11 | 12.93 (6.46 - 23.14) ^b^ | 18 | 7.82 (4.64 - 12.36) ^b^ | 12 | 12.66 (6.54 - 22.12) ^b^ | 5 | 25.93 (8.42 - 60.51) ^b^ | 46 | 10.72 (7.85 - 14.3) ^b^ |
| Diabetes Mellitus | 10 | 3.95 (1.9 - 7.27) ^b^ | 25 | 2.62 (1.7 - 3.87) ^b^ | 31 | 3.23 (2.2 - 4.59) ^b^ | 23 | 5.13 (3.25 - 7.7) ^b^ | 89 | 3.4 (2.73 - 4.19) ^b^ |
| Alzheimer’s (ICD-9 and 10 only) | 3 | 0.71 (0.15 - 2.08) | 22 | 1.43 (0.9 - 2.17) | 23 | 2.06 (1.31 - 3.09) ^b^ | 14 | 3.51 (1.92 - 5.88) ^b^ | 62 | 1.78 (1.37 - 2.29) ^b^ |
| Diseases of Heart | 176 | 4.2 (3.6 - 4.87) ^b^ | 431 | 3.39 (3.07 - 3.72) ^b^ | 336 | 3.66 (3.28 - 4.08) ^b^ | 202 | 5.23 (4.53 - 6) ^b^ | 1,145 | 3.82 (3.6 - 4.05) ^b^ |
| Hypertension without Heart Disease | 5 | 3.42 (1.11 - 7.98) ^b^ | 13 | 2.88 (1.53 - 4.92) ^b^ | 15 | 3.72 (2.08 - 6.14) ^b^ | 7 | 4.35 (1.75 - 8.97) ^b^ | 40 | 3.44 (2.46 - 4.69) ^b^ |
| Cerebrovascular Diseases | 27 | 3.49 (2.3 - 5.07) ^b^ | 87 | 3.68 (2.95 - 4.54) ^b^ | 67 | 4.29 (3.33 - 5.45) ^b^ | 36 | 6.14 (4.3 - 8.5) ^b^ | 217 | 4.11 (3.58 - 4.69) ^b^ |
| Atherosclerosis | 1 | 3.79 (0.1 - 21.12) | 4 | 3.64 (0.99 - 9.31) | 5 | 10.35 (3.36 - 24.16) ^b^ | 0 | 0 (0 - 0) | 10 | 5.41 (2.6 - 9.96) ^b^ |
| Aortic Aneurysm and Dissection | 0 | 0 (0 - 6.55) | 13 | 6.6 (3.51 - 11.28) ^b^ | 8 | 5.87 (2.53 - 11.57) ^b^ | 2 | 16.84 (2.04 - 60.83) ^b^ | 23 | 5.73 (3.63 - 8.6) ^b^ |
| Other Diseases of Arteries, Arterioles, Capillaries | 5 | 9.67 (3.14 - 22.58) ^b^ | 7 | 7.24 (2.91 - 14.91) ^b^ | 2 | 5.56 (0.67 - 20.08) | 1 | 11.18 (0.28 - 62.31) | 15 | 7.76 (4.34 - 12.8) ^b^ |
| Pneumonia and Influenza | 15 | 2.55 (1.43 - 4.21) ^b^ | 54 | 2.81 (2.11 - 3.66) ^b^ | 34 | 3.09 (2.14 - 4.32) ^b^ | 29 | 5.73 (3.84 - 8.23) ^b^ | 132 | 3.2 (2.68 - 3.8) ^b^ |
| Chronic Obstructive Pulmonary Disease and Allied Cond | 57 | 3.37 (2.55 - 4.37) ^b^ | 180 | 3.17 (2.72 - 3.67) ^b^ | 163 | 4.25 (3.62 - 4.95) ^b^ | 103 | 6.68 (5.45 - 8.1) ^b^ | 503 | 3.95 (3.61 - 4.31) ^b^ |
| Stomach and Duodenal Ulcers | 4 | 6.41 (1.75 - 16.41) ^b^ | 8 | 4.28 (1.85 - 8.43) ^b^ | 3 | 3.34 (0.69 - 9.75) | 1 | 19.41 (0.49 - 108.13) | 16 | 4.64 (2.65 - 7.54) ^b^ |
| Chronic Liver Disease and Cirrhosis | 6 | 6.32 (2.32 - 13.76) ^b^ | 30 | 9.86 (6.66 - 14.08) ^b^ | 17 | 9.37 (5.46 - 15) ^b^ | 5 | 30.75 (9.98 - 71.76) ^b^ | 58 | 9.72 (7.38 - 12.56) ^b^ |
| Nephritis, Nephrotic Syndrome and Nephrosis | 8 | 1.91 (0.82 - 3.75) | 25 | 1.72 (1.11 - 2.54) ^b^ | 30 | 3.54 (2.39 - 5.06) ^b^ | 12 | 5.02 (2.6 - 8.78) ^b^ | 75 | 2.53 (1.99 - 3.18) ^b^ |
| Complications of Pregnancy, Childbirth, Puerperium | 0 | 0 (0 - 4067.39) | 1 | 457.03 (11.57 - 2546.39) ^b^ | 0 | 0 (0 - 0) | 0 | 0 (0 - 0) | 1 | 323.1 (8.18 - 1800.21) ^b^ |
| Congenital Anomalies | 0 | 0 (0 - 53.59) | 1 | 3.18 (0.08 - 17.71) | 1 | 2.47 (0.06 - 13.78) | 0 | 0 (0 - 0) | 2 | 2.54 (0.31 - 9.17) |
| Certain Conditions Originating in Perinatal Period | 0 | 0 (0 - 0) | 0 | 0 (0 - 0) | 0 | 0 (0 - 0) | 0 | 0 (0 - 0) | 0 | 0 (0 - 0) |
| Symptoms, Signs, and Ill-Defined Conditions | 9 | 5.17 (2.36 - 9.81) ^b^ | 21 | 2.96 (1.83 - 4.53) ^b^ | 24 | 3.23 (2.07 - 4.81) ^b^ | 10 | 3.29 (1.58 - 6.05) ^b^ | 64 | 3.32 (2.55 - 4.24) ^b^ |
| Accidents and Adverse Effects | 32 | 7.4 (5.06 - 10.44) ^b^ | 59 | 4.41 (3.35 - 5.68) ^b^ | 44 | 6.36 (4.62 - 8.53) ^b^ | 17 | 11.06 (6.44 - 17.7) ^b^ | 152 | 5.81 (4.92 - 6.81) ^b^ |
| Suicide and Self-Inflicted Injury | 18 | 12.52 (7.42 - 19.79) ^b^ | 29 | 8.67 (5.81 - 12.46) ^b^ | 15 | 10.06 (5.63 - 16.59) ^b^ | 4 | 7.16 (1.95 - 18.34) ^b^ | 66 | 9.66 (7.47 - 12.29) ^b^ |
| Homicide and Legal Intervention | 2 | 12.36 (1.5 - 44.65) ^b^ | 5 | 18.14 (5.89 - 42.34) ^b^ | 1 | 451.29 (11.43 - 2514.44) ^b^ | 0 | 0 (0 - 0) | 8 | 18.2 (7.86 - 35.86) ^b^ |
| Other Cause of Death | 72 | 3.36 (2.63 - 4.24) ^b^ | 209 | 2.88 (2.51 - 3.3) ^b^ | 184 | 3.62 (3.11 - 4.18) ^b^ | 108 | 5.68 (4.66 - 6.86) ^b^ | 573 | 3.5 (3.22 - 3.8) ^b^ |

**1** number of cancer patients who died due to each cause of death **2** 95% Confidence interval **b** P-value < 0.05

| **Supplementary Table 14.** Standardized-mortality ratios (SMRs) for each cause of death following laryngeal cancer diagnosis in patients who received radiotherapy. | | | | | | | | | | |
| --- | --- | --- | --- | --- | --- | --- | --- | --- | --- | --- |
| **Cause of death** | <1 year | | 1-5 years | | 5-10 years | | >10 years | | Total | |
|  | Observed^1^ | SMR (95% CI^2^) | Observed^1^ | SMR (95% CI^2^) | Observed^1^ | SMR (95% CI^2^) | Observed^1^ | SMR (95% CI^2^) | Observed^1^ | SMR (95% CI^2^) |
| All causes of death | 3,825 | 7.99 (7.74 - 8.25) ^b^ | 9,020 | 7.5 (7.34 - 7.65) ^b^ | 3,981 | 5.4 (5.23 - 5.57) ^b^ | 1,786 | 7.18 (6.85 - 7.52) ^b^ | 18,612 | 6.97 (6.87 - 7.08) ^b^ |
| Laryngeal cancer | 1,792 | 13.69 (13.06 - 14.34) ^b^ | 3,518 | 17.85 (17.27 - 18.45) ^b^ | 606 | 8.93 (8.24 - 9.67) ^b^ | 178 | 10.47 (8.99 - 12.12) ^b^ | 6,094 | 14.76 (14.39 - 15.14) ^b^ |
| Other non-laryngeal cancer causes of death | 1,078 | 8.41 (7.91 - 8.92) ^b^ | 2,897 | 8.87 (8.55 - 9.2) ^b^ | 1,340 | 6.7 (6.34 - 7.07) ^b^ | 572 | 8.79 (8.08 - 9.54) ^b^ | 5,887 | 8.18 (7.97 - 8.39) ^b^ |
| All non-cancer causes of death | 955 | 4.35 (4.08 - 4.63) ^b^ | 2,605 | 3.83 (3.69 - 3.98) ^b^ | 2,035 | 4.33 (4.15 - 4.52) ^b^ | 1,036 | 6.21 (5.84 - 6.6) ^b^ | 6,631 | 4.32 (4.21 - 4.42) ^b^ |
| In situ, benign or unknown behavior neoplasm | 9 | 3.47 (1.59 - 6.59) ^b^ | 47 | 6.19 (4.55 - 8.24) ^b^ | 19 | 4.4 (2.65 - 6.87) ^b^ | 15 | 6.86 (3.84 - 11.31) ^b^ | 90 | 5.39 (4.34 - 6.63) ^b^ |
| Tuberculosis | 0 | 0 (0 - 207.91) | 1 | 30.58 (0.77 - 170.36) | 0 | 0 (0 - 0) | 0 | 0 (0 - 0) | 1 | 19.82 (0.5 - 110.44) |
| Syphilis | 0 | 0 (0 - 0) | 0 | 0 (0 - 0) | 0 | 0 (0 - 0) | 0 | 0 (0 - 0) | 0 | 0 (0 - 0) |
| Septicemia | 33 | 8.68 (5.97 - 12.19) ^b^ | 59 | 6.69 (5.09 - 8.63) ^b^ | 32 | 5.08 (3.47 - 7.16) ^b^ | 22 | 6.85 (4.29 - 10.36) ^b^ | 146 | 6.59 (5.57 - 7.75) ^b^ |
| Other Infectious and Parasitic Diseases including HIV | 27 | 11.5 (7.58 - 16.74) ^b^ | 44 | 7.99 (5.8 - 10.72) ^b^ | 31 | 8.89 (6.04 - 12.63) ^b^ | 11 | 7.56 (3.77 - 13.52) ^b^ | 113 | 8.83 (7.28 - 10.62) ^b^ |
| Diabetes Mellitus | 20 | 4.36 (2.66 - 6.73) ^b^ | 67 | 4.54 (3.52 - 5.77) ^b^ | 49 | 4.04 (2.99 - 5.34) ^b^ | 32 | 5.67 (3.88 - 8.01) ^b^ | 168 | 4.53 (3.87 - 5.27) ^b^ |
| Alzheimer’s (ICD-9 and 10 only) | 6 | 0.81 (0.3 - 1.76) | 36 | 1.31 (0.92 - 1.81) | 45 | 2.33 (1.7 - 3.11) ^b^ | 26 | 3.53 (2.31 - 5.17) ^b^ | 113 | 1.83 (1.51 - 2.2) ^b^ |
| Diseases of Heart | 354 | 4.61 (4.14 - 5.11) ^b^ | 863 | 3.73 (3.49 - 3.99) ^b^ | 634 | 4.08 (3.77 - 4.41) ^b^ | 330 | 6 (5.37 - 6.68) ^b^ | 2,181 | 4.21 (4.03 - 4.39) ^b^ |
| Hypertension without Heart Disease | 15 | 4.18 (2.34 - 6.9) ^b^ | 33 | 3.11 (2.14 - 4.36) ^b^ | 25 | 2.84 (1.84 - 4.2) ^b^ | 14 | 5.36 (2.93 - 9) ^b^ | 87 | 3.4 (2.72 - 4.19) ^b^ |
| Cerebrovascular Diseases | 35 | 2.48 (1.73 - 3.45) ^b^ | 180 | 4.11 (3.53 - 4.75) ^b^ | 141 | 4.75 (3.99 - 5.6) ^b^ | 71 | 6.59 (5.14 - 8.31) ^b^ | 427 | 4.34 (3.94 - 4.77) ^b^ |
| Atherosclerosis | 2 | 2.93 (0.35 - 10.58) | 7 | 3.83 (1.54 - 7.88) ^b^ | 8 | 11.4 (4.92 - 22.47) ^b^ | 0 | 0 (0 - 0) | 17 | 5.29 (3.08 - 8.47) ^b^ |
| Aortic Aneurysm and Dissection | 6 | 4.08 (1.5 - 8.88) ^b^ | 23 | 4.68 (2.97 - 7.02) ^b^ | 18 | 5.46 (3.24 - 8.63) ^b^ | 7 | 10.5 (4.22 - 21.64) ^b^ | 54 | 5.22 (3.92 - 6.81) ^b^ |
| Other Diseases of Arteries, Arterioles, Capillaries | 9 | 8.58 (3.92 - 16.28) ^b^ | 19 | 6.75 (4.07 - 10.54) ^b^ | 15 | 8.15 (4.56 - 13.44) ^b^ | 2 | 15.97 (1.93 - 57.68) ^b^ | 45 | 7.72 (5.63 - 10.33) ^b^ |
| Pneumonia and Influenza | 42 | 4.64 (3.34 - 6.27) ^b^ | 102 | 4.09 (3.34 - 4.97) ^b^ | 61 | 4.1 (3.13 - 5.26) ^b^ | 44 | 8.04 (5.84 - 10.79) ^b^ | 249 | 4.58 (4.03 - 5.19) ^b^ |
| Chronic Obstructive Pulmonary Disease and Allied Cond | 137 | 4.37 (3.67 - 5.16) ^b^ | 411 | 4.17 (3.77 - 4.59) ^b^ | 342 | 5.15 (4.62 - 5.72) ^b^ | 165 | 7.46 (6.36 - 8.68) ^b^ | 1,055 | 4.83 (4.54 - 5.13) ^b^ |
| Stomach and Duodenal Ulcers | 5 | 7.55 (2.45 - 17.62) ^b^ | 8 | 3.81 (1.65 - 7.51) ^b^ | 4 | 2.78 (0.76 - 7.12) | 2 | 4.65 (0.56 - 16.78) | 19 | 4.1 (2.47 - 6.41) ^b^ |
| Chronic Liver Disease and Cirrhosis | 17 | 11.87 (6.91 - 19) ^b^ | 43 | 10.65 (7.71 - 14.34) ^b^ | 32 | 11.89 (8.13 - 16.78) ^b^ | 9 | 16.14 (7.38 - 30.64) ^b^ | 101 | 11.58 (9.43 - 14.07) ^b^ |
| Nephritis, Nephrotic Syndrome and Nephrosis | 16 | 2.52 (1.44 - 4.1) ^b^ | 51 | 2.34 (1.74 - 3.07) ^b^ | 49 | 3.33 (2.46 - 4.4) ^b^ | 20 | 5.09 (3.11 - 7.86) ^b^ | 136 | 2.9 (2.44 - 3.44) ^b^ |
| Complications of Pregnancy, Childbirth, Puerperium | 0 | 0 (0 - 0) | 0 | 0 (0 - 0) | 0 | 0 (0 - 0) | 0 | 0 (0 - 0) | 0 | 0 (0 - 0) |
| Congenital Anomalies | 0 | 0 (0 - 35.39) | 2 | 4.74 (0.57 - 17.12) | 2 | 3.9 (0.47 - 14.11) | 1 | 133.48 (3.38 - 743.72) ^b^ | 5 | 4.78 (1.55 - 11.16) ^b^ |
| Certain Conditions Originating in Perinatal Period | 0 | 0 (0 - 0) | 0 | 0 (0 - 0) | 0 | 0 (0 - 0) | 0 | 0 (0 - 0) | 0 | 0 (0 - 0) |
| Symptoms, Signs, and Ill-Defined Conditions | 16 | 4.72 (2.7 - 7.67) ^b^ | 36 | 2.94 (2.06 - 4.07) ^b^ | 37 | 2.98 (2.1 - 4.11) ^b^ | 17 | 5.78 (3.37 - 9.26) ^b^ | 106 | 3.42 (2.8 - 4.14) ^b^ |
| Accidents and Adverse Effects | 48 | 6.35 (4.68 - 8.42) ^b^ | 111 | 4.65 (3.83 - 5.6) ^b^ | 89 | 6.05 (4.86 - 7.45) ^b^ | 37 | 9.61 (6.77 - 13.25) ^b^ | 285 | 5.7 (5.06 - 6.4) ^b^ |
| Suicide and Self-Inflicted Injury | 24 | 13.61 (8.72 - 20.25) ^b^ | 47 | 12.46 (9.16 - 16.57) ^b^ | 24 | 12.83 (8.22 - 19.09) ^b^ | 6 | 9.03 (3.31 - 19.65) ^b^ | 101 | 12.51 (10.19 - 15.21) ^b^ |
| Homicide and Legal Intervention | 1 | 4.7 (0.12 - 26.21) | 7 | 12.57 (5.05 - 25.9) ^b^ | 3 | 13.41 (2.76 - 39.18) ^b^ | 1 | 2.22 (0.06 - 12.39) | 12 | 8.32 (4.3 - 14.53) ^b^ |
| Other Cause of Death | 133 | 3.39 (2.84 - 4.02) ^b^ | 408 | 3.19 (2.89 - 3.52) ^b^ | 375 | 3.96 (3.57 - 4.38) ^b^ | 204 | 5.47 (4.74 - 6.27) ^b^ | 1,120 | 3.75 (3.53 - 3.97) ^b^ |

**1** number of cancer patients who died due to each cause of death **2** 95% Confidence interval **b** P-value < 0.05

| **Supplementary Table 15.** Standardized-mortality ratios (SMRs) for each cause of death following laryngeal cancer diagnosis in patients who received chemotherapy. | | | | | | | | | | |
| --- | --- | --- | --- | --- | --- | --- | --- | --- | --- | --- |
| **Cause of death** | <1 year | | 1-5 years | | 5-10 years | | >10 years | | Total | |
|  | Observed^1^ | SMR (95% CI^2^) | Observed^1^ | SMR (95% CI^2^) | Observed^1^ | SMR (95% CI^2^) | Observed^1^ | SMR (95% CI^2^) | Observed^1^ | SMR (95% CI^2^) |
| All causes of death | 2,378 | 16.42 (15.76 - 17.09) ^b^ | 4,462 | 15.73 (15.28 - 16.2) ^b^ | 1,376 | 9.54 (9.04 - 10.06) ^b^ | 493 | 12.5 (11.42 - 13.66) ^b^ | 8,709 | 14.23 (13.93 - 14.53) ^b^ |
| Laryngeal cancer | 1,198 | 20.9 (19.73 - 22.12) ^b^ | 2,024 | 26.88 (25.72 - 28.07) ^b^ | 276 | 11.9 (10.54 - 13.39) ^b^ | 70 | 16.95 (13.21 - 21.41) ^b^ | 3,568 | 22.31 (21.58 - 23.05) ^b^ |
| Other non-laryngeal cancer causes of death | 719 | 15.4 (14.29 - 16.57) ^b^ | 1,576 | 16.79 (15.97 - 17.64) ^b^ | 499 | 10.74 (9.82 - 11.73) ^b^ | 176 | 13.58 (11.65 - 15.74) ^b^ | 2,970 | 14.85 (14.32 - 15.4) ^b^ |
| All non-cancer causes of death | 461 | 11.29 (10.28 - 12.37) ^b^ | 862 | 7.54 (7.04 - 8.06) ^b^ | 601 | 8.05 (7.42 - 8.72) ^b^ | 247 | 11.06 (9.72 - 12.52) ^b^ | 2,171 | 8.61 (8.25 - 8.98) ^b^ |
| In situ, benign or unknown behavior neoplasm | 5 | 10.95 (3.55 - 25.55) ^b^ | 19 | 17.91 (10.78 - 27.97) ^b^ | 7 | 23.39 (9.41 - 48.2) ^b^ | 1 | 45.05 (1.14 - 251.03) ^b^ | 32 | 17.4 (11.9 - 24.57) ^b^ |
| Tuberculosis | 0 | 0 (0 - 207.91) | 1 | 30.58 (0.77 - 170.36) | 0 | 0 (0 - 0) | 0 | 0 (0 - 0) | 1 | 19.82 (0.5 - 110.44) |
| Syphilis | 0 | 0 (0 - 0) | 0 | 0 (0 - 0) | 0 | 0 (0 - 0) | 0 | 0 (0 - 0) | 0 | 0 (0 - 0) |
| Septicemia | 22 | 22.47 (14.08 - 34.02) ^b^ | 24 | 14.03 (8.99 - 20.88) ^b^ | 11 | 10.71 (5.34 - 19.16) ^b^ | 6 | 18.53 (6.8 - 40.32) ^b^ | 63 | 15.59 (11.98 - 19.95) ^b^ |
| Other Infectious and Parasitic Diseases including HIV | 17 | 38.88 (22.65 - 62.25) ^b^ | 19 | 21.47 (12.93 - 33.54) ^b^ | 8 | 39.58 (17.09 - 77.99) ^b^ | 1 | 288.43 (7.3 - 1607.05) ^b^ | 45 | 29.46 (21.49 - 39.42) ^b^ |
| Diabetes Mellitus | 6 | 8.91 (3.27 - 19.39) ^b^ | 21 | 13.21 (8.18 - 20.19) ^b^ | 8 | 10.57 (4.56 - 20.82) ^b^ | 6 | 24.38 (8.95 - 53.06) ^b^ | 41 | 12.55 (9.01 - 17.03) ^b^ |
| Alzheimer’s (ICD-9 and 10 only) | 1 | 1.68 (0.04 - 9.35) | 4 | 1.77 (0.48 - 4.53) | 3 | 1.87 (0.39 - 5.47) | 3 | 7.1 (1.46 - 20.76) ^b^ | 11 | 2.25 (1.13 - 4.03) ^b^ |
| Diseases of Heart | 178 | 13.79 (11.84 - 15.97) ^b^ | 269 | 7.72 (6.82 - 8.7) ^b^ | 168 | 6.93 (5.92 - 8.06) ^b^ | 72 | 9.89 (7.74 - 12.46) ^b^ | 687 | 8.66 (8.03 - 9.34) ^b^ |
| Hypertension without Heart Disease | 8 | 14.28 (6.16 - 28.13) ^b^ | 11 | 8.66 (4.32 - 15.49) ^b^ | 7 | 8.83 (3.55 - 18.19) ^b^ | 4 | 16.99 (4.63 - 43.49) ^b^ | 30 | 10.49 (7.08 - 14.98) ^b^ |
| Cerebrovascular Diseases | 13 | 5.17 (2.75 - 8.84) ^b^ | 54 | 6.38 (4.79 - 8.32) ^b^ | 43 | 8.18 (5.92 - 11.02) ^b^ | 23 | 10.39 (6.59 - 15.59) ^b^ | 133 | 7.21 (6.04 - 8.54) ^b^ |
| Atherosclerosis | 1 | 10.55 (0.27 - 58.78) | 1 | 3.41 (0.09 - 19.02) | 3 | 22.62 (4.66 - 66.09) ^b^ | 0 | 0 (0 - 0) | 5 | 9.61 (3.12 - 22.42) ^b^ |
| Aortic Aneurysm and Dissection | 4 | 9.55 (2.6 - 24.44) ^b^ | 5 | 3.58 (1.16 - 8.36) ^b^ | 7 | 8.11 (3.26 - 16.71) ^b^ | 3 | 12.33 (2.54 - 36.03) ^b^ | 19 | 6.5 (3.92 - 10.16) ^b^ |
| Other Diseases of Arteries, Arterioles, Capillaries | 4 | 24.55 (6.69 - 62.86) ^b^ | 3 | 5.63 (1.16 - 16.45) ^b^ | 8 | 19.34 (8.35 - 38.11) ^b^ | 0 | 0 (0 - 0) | 15 | 13.52 (7.57 - 22.3) ^b^ |
| Pneumonia and Influenza | 17 | 8.33 (4.85 - 13.34) ^b^ | 40 | 8.14 (5.81 - 11.08) ^b^ | 25 | 6.2 (4.01 - 9.15) ^b^ | 14 | 11.06 (6.04 - 18.55) ^b^ | 96 | 7.83 (6.35 - 9.57) ^b^ |
| Chronic Obstructive Pulmonary Disease and Allied Cond | 46 | 6.85 (5.01 - 9.13) ^b^ | 138 | 6.85 (5.75 - 8.09) ^b^ | 112 | 8.74 (7.19 - 10.51) ^b^ | 38 | 15.62 (11.06 - 21.45) ^b^ | 334 | 7.93 (7.1 - 8.83) ^b^ |
| Stomach and Duodenal Ulcers | 3 | 36.7 (7.57 - 107.25) ^b^ | 4 | 38.76 (10.56 - 99.24) ^b^ | 0 | 0 (0 - 0) | 0 | 0 (0 - 0) | 7 | 37.85 (15.22 - 77.98) ^b^ |
| Chronic Liver Disease and Cirrhosis | 12 | 23.72 (12.26 - 41.44) ^b^ | 17 | 14.14 (8.24 - 22.64) ^b^ | 9 | 12.21 (5.58 - 23.18) ^b^ | 4 | 9.33 (2.54 - 23.9) ^b^ | 42 | 14.62 (10.53 - 19.76) ^b^ |
| Nephritis, Nephrotic Syndrome and Nephrosis | 4 | 7.98 (2.17 - 20.42) ^b^ | 13 | 7.06 (3.76 - 12.07) ^b^ | 6 | 4.05 (1.49 - 8.82) ^b^ | 5 | 16.38 (5.32 - 38.22) ^b^ | 28 | 6.78 (4.5 - 9.8) ^b^ |
| Complications of Pregnancy, Childbirth, Puerperium | 0 | 0 (0 - 4067.39) | 1 | 457.03 (11.57 - 2546.39) ^b^ | 0 | 0 (0 - 0) | 0 | 0 (0 - 0) | 1 | 323.1 (8.18 - 1800.21) ^b^ |
| Congenital Anomalies | 1 | 27.66 (0.7 - 154.13) | 1 | 9.31 (0.24 - 51.88) | 1 | 9.26 (0.23 - 51.6) | 1 | 133.48 (3.38 - 743.72) ^b^ | 4 | 15.44 (4.21 - 39.54) ^b^ |
| Certain Conditions Originating in Perinatal Period | 0 | 0 (0 - 0) | 0 | 0 (0 - 0) | 0 | 0 (0 - 0) | 0 | 0 (0 - 0) | 0 | 0 (0 - 0) |
| Symptoms, Signs, and Ill-Defined Conditions | 10 | 14.33 (6.87 - 26.35) ^b^ | 19 | 8.22 (4.95 - 12.84) ^b^ | 14 | 10.96 (5.99 - 18.4) ^b^ | 2 | 11.42 (1.38 - 41.25) ^b^ | 45 | 10.09 (7.36 - 13.5) ^b^ |
| Accidents and Adverse Effects | 27 | 11.32 (7.46 - 16.47) ^b^ | 47 | 7.58 (5.57 - 10.08) ^b^ | 33 | 7.75 (5.34 - 10.88) ^b^ | 10 | 7.07 (3.39 - 13.01) ^b^ | 117 | 8.21 (6.79 - 9.83) ^b^ |
| Suicide and Self-Inflicted Injury | 9 | 23.23 (10.62 - 44.1) ^b^ | 18 | 21.78 (12.91 - 34.43) ^b^ | 8 | 20.21 (8.72 - 39.82) ^b^ | 2 | 7.35 (0.89 - 26.55) | 37 | 19.66 (13.84 - 27.1) ^b^ |
| Homicide and Legal Intervention | 0 | 0 (0 - 95.96) | 0 | 0 (0 - 17.29) | 2 | 9.03 (1.09 - 32.61) ^b^ | 1 | 2.22 (0.06 - 12.39) | 3 | 3.25 (0.67 - 9.5) |
| Other Cause of Death | 73 | 9.57 (7.5 - 12.03) ^b^ | 133 | 6 (5.02 - 7.11) ^b^ | 118 | 8.61 (7.13 - 10.31) ^b^ | 51 | 11.08 (8.25 - 14.57) ^b^ | 375 | 7.8 (7.03 - 8.63) ^b^ |

**1** number of cancer patients who died due to each cause of death **2** 95% Confidence interval **b** P-value < 0.05
